# Dissecting the brown adipogenic regulatory network using integrative genomics

**DOI:** 10.1038/srep42130

**Published:** 2017-02-09

**Authors:** Rachana N. Pradhan, Johannes J. Bues, Vincent Gardeux, Petra C. Schwalie, Daniel Alpern, Wanze Chen, Julie Russeil, Sunil K. Raghav, Bart Deplancke

**Affiliations:** 1 Institute of Bioengineering, École Polytechnique Fédérale de Lausanne (EPFL), CH-1015 Lausanne, Switzerland; 2 Swiss Institute of Bioinformatics, CH-1015 Lausanne, Switzerland; 3 ILS Bhubhaneshwar, Orissa, India

## Abstract

Brown adipocytes regulate energy expenditure via mitochondrial uncoupling, which makes them attractive therapeutic targets to tackle obesity. However, the regulatory mechanisms underlying brown adipogenesis are still poorly understood. To address this, we profiled the transcriptome and chromatin state during mouse brown fat cell differentiation, revealing extensive gene expression changes and chromatin remodeling, especially during the first day post-differentiation. To identify putatively causal regulators, we performed transcription factor binding site overrepresentation analyses in active chromatin regions and prioritized factors based on their expression correlation with the *bona-fide* brown adipogenic marker *Ucp1* across multiple mouse and human datasets. Using loss-of-function assays, we evaluated both the phenotypic effect as well as the transcriptomic impact of several putative regulators on the differentiation process, uncovering ZFP467, HOXA4 and Nuclear Factor I A (NFIA) as novel transcriptional regulators. Of these, NFIA emerged as the regulator yielding the strongest molecular and cellular phenotypes. To examine its regulatory function, we profiled the genomic localization of NFIA, identifying it as a key early regulator of terminal brown fat cell differentiation.

Given the rise in the obesity pandemic and its associated pathologies, there is an active interest in the pursuit of pathways and therapeutic targets to regulate metabolic balance. With the recent discovery of metabolically active brown fat and brown-in-white fat depots in adult humans^1^,^2^,^3^, the field is extensively exploring ways to maximize energy expenditure over storage via activation of the brown adipose tissue (BAT). In contrast to its energy-storing counterpart, the white adipocyte, the brown adipocyte is capable of converting fat reserves directly to heat. This heat generating process, termed thermogenesis, is mediated by the mitochondrial uncoupling protein 1 (UCP1), the hallmark of a differentiated and metabolically active brown adipocyte^4^.

The terminal differentiation program of white adipocytes has been studied extensively, given the availability of several *in vitro* cell culture models (reviewed in ref. 5), while the regulatory events mediating brown fat cell (BFC) differentiation are still poorly characterized (reviewed in ref. 6). Indeed, several studies have thoroughly investigated the general white adipocyte differentiation program, leading to the identification of the master regulator PPARγ^7^, other core regulators such as C/EBPs^8^, RXRγ^9^, ZEB1^10^, and Kruppel-like factors^11^,^12^,^13^,^14^. Additionally, several chromatin modification and transcription (co-) factor (TF) binding maps have been generated, providing insights into chromatin state dynamics during differentiation and the regulatory components mediating it^15^,^16^,^17^,^18^,^19^.

Efforts are now being directed toward generating brown adipocyte cell models able to faithfully mimic the differentiation process, both in murine^20^,^21^ and human systems^22^,^23^. This has allowed the investigation and subsequent identification of early regulators of brown adipocyte commitment such as PRDM16^24^, EBF2^25^, PLAC8^26^ along with core thermogenic regulators such as PGC1α^27^, PPARα^28^ and more recently KLF11^29^ and ZFP516^30^. However, a comprehensive spatio-temporal regulatory analysis of the brown fat differentiation process is still missing.

In this study, we leveraged the power of transcriptomics to categorize differentially expressed genes showing similar gene expression trends during BFC differentiation and selected transcriptional regulators whose expression was highly correlated with that of the *bona fide* BFC marker *Ucp1*. We then confirmed that this molecular relation holds true *in vivo*, using data from brown fat of tens of mice included in the mouse genetic reference panel (BXD)^31^ as well as partially extends to humans, as revealed by data retrieved from tens of clonal cell lines derived from BAT^22^. Furthermore, using high-resolution, dynamic profiles of acetylation on lysine 27 of the H3 histone (H3K27ac), we determined which of the candidate regulators were likely to bind active regulatory regions^32^ by performing transcription factor binding site (TFBS) enrichment analyses. Finally, we assessed the transcriptomic and phenotypic impact of knocking down the main candidates, implicating the TFs NFIA, HOXA4 and ZFP467 for the first time in brown adipogenesis. Subsequent ChIP-seq experiments revealed NFIA to be a key activator in the brown adipogenic gene regulatory network (GRN). Together, our study provides to our knowledge the first comprehensive overview of transcriptomic and epigenomic changes underlying BFC differentiation and highlights novel regulators of this poorly characterized, yet biomedically important differentiation process.

## Results

### *In vitro* murine brown adipogenesis mimics the *in vivo* process

**Figure 1:**
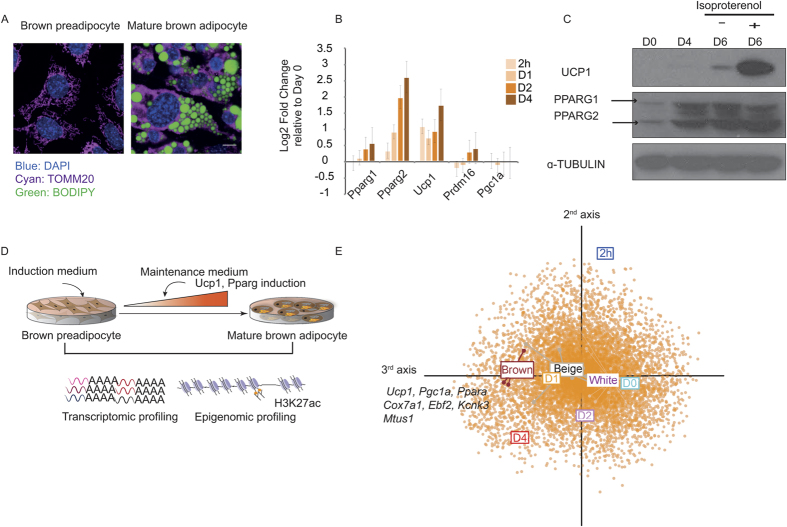
Functional validation of an *in vitro* differentiating murine brown preadipocyte cell line. (**A**) Fluorescent staining and confocal microscopy images of brown preadipocytes versus differentiated mature brown adipocytes. Blue DAPI stains indicate nuclei, green BODIPY stains lipid droplets and Cyan TOM20 specific stain identifies mitochondria. Scale bar: 10 um. (**B**) Gene expression levels of the brown fat marker genes *Pparg1, Pparg2, Ucp1, Prdm16* and *Pgc1a*, as measured using quantitative PCR, represented as log2 fold change relative to Day 0 (n = 3, data are presented as mean ± SEM). (**C**) Protein levels of UCP1, PPARγ (both PPARγ1 and PPARγ2 isoforms indicated) and endogenous control α-TUBULIN at different time points during differentiation, specifically, Day 0, Day 4, and Day 6 with and without treatment with the β-adrenergic stimulator, isoproterenol. Note that this image has been cropped from the film in Supplementary Fig. 4A. (**D**) Schematic representation of the experimental strategy and the quantitative measurements of the transcriptome and the epigenome during differentiation. (**E**) Correspondence analysis of *ex vivo* differentiated murine brown fat cells and *in vitro* (IBA) differentiated samples. The third axis contains genes explaining the differences between white versus brown cells (see Methods).

To facilitate the temporal characterization of BFC differentiation, we used an immortalized brown preadipocyte cell line (IBA)^20^ (see Methods). To validate the functionality of our IBA cell line, we evaluated the differentiation process by stimulating confluent cells with a brown-specific adipogenic cocktail (see Methods). We selected six time points corresponding to different stages of differentiation: confluence (Day 0), 2 hours post induction (2 h), maturing adipocytes (Day 1 and Day 2), and mature adipocytes (Day 4 and Day 6). To assess whether the process of differentiation mimicked the *in vivo* system, we first looked for phenotypic evidence in the form of lipid accumulation and mitochondrial remodeling^33^. Indeed, upon inducing the brown preadipocytes to mature brown fat cells, we clearly observed such phenotypic alterations, indicative of differentiation (Fig. 1A). Next, we performed gene expression profiling of key brown fat and adipogenic markers and observed a marked increase in mRNA levels of the adipogenic master regulator *Pparg* along with the brown fat marker gene *Ucp1*, over the course of differentiation (Fig. 1B). In coherence with the observed gene expression profiles, protein expression measurements showed an increase in both PPARγ and UCP1 levels. Moreover, the mature brown adipocyte at Day 6 responded to the beta-adrenergic stimulator isoproterenol, showing a massive induction in UCP1 levels, reflective of a potent activation of the brown thermogenic program^34^ (Fig. 1C). These lines of evidence indicate that our brown preadipocyte cell line has the predisposition to differentiate into mature brown adipocytes that are amenable to metabolic activation. We then chose to study the underlying terminal BFC differentiation process until the formation of mature brown adipocytes (by Day 4), given that our primary interest was to identify TFs that induce an increase in *Ucp1* expression.

We next aimed at exploring global transcriptome changes using mRNA-seq during the differentiation of brown preadipocytes to mature brown adipocytes (Day 0, 2 h, Day 1, Day 2 and Day 4) (Fig. 1D). We found 964 out of a total of 11,859 detected genes to be differentially expressed, accounting for the dynamic changes during differentiation (absolute log2 fold change >= 2, FDR <0.01, see Methods and Fig. S1A). Given that our study measures for the first time genome-wide gene expression changes during BFC differentiation, we aimed to validate the transcriptomic outcome of our *in vitro* differentiation system. To do so, we compared the obtained gene expression profiles with microarray data available from *ex vivo* differentiated cells derived from murine fat pads, including the subcutaneous white adipose depot and the interscapular brown adipose tissue^21^. Using correspondence analysis (see Methods) we observed that Day 1, Day 2 and Day 4 brown adipocytes resemble the *ex vivo* differentiated clonal cells classified as phenotypically brown and beige, indicated by the closeness of their projection along the same axis (3^rd^ axis, Fig. 1E). In contrast to the maturing brown adipocytes, brown preadipocytes at Day 0 and those immediately post induction (2 h) were closer to the inguinal white adipocyte-derived *ex vivo* differentiated cells (Fig. 1E). In particular, key marker genes of brown fat differentiation such as *Ucp1*, key regulators including *Ppara, Ebf2* and *Pgc1a* and recently discovered thermogenic markers, *Kckn3* and *Mtus1*^23^ were some of the drivers of the observed differences. This is indicated by their increased expression in brown over white cells as well as in Day 4 mature brown adipocytes over Day 0 preadipocytes. These results demonstrate that Day 4 mature brown adipocytes globally resemble *ex vivo* differentiated BAT-derived cells and further support the notion that our *in vitro* system is representative for BFC differentiation.

### Transcriptomic analysis during BFC differentiation reveals four distinct gene expression patterns

**Figure 2:**
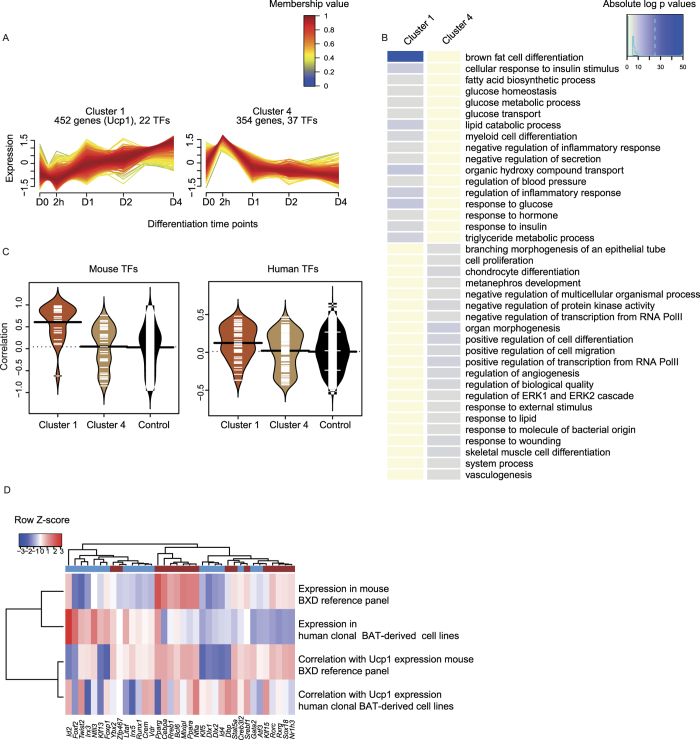
Transcriptome-based identification of putative transcriptional regulators of brown adipogenesis. (**A**) Fuzzy c-means clusters depicting putative positive regulators (Cluster 1, increase in expression) and early stage-specific regulators (Cluster 4, spike in expression at 2 h) of differentiation with red lines indicating members strongly associated with the cluster. (**B**) Gene ontology (GO) terms associated with genes from Cluster 1 and Cluster 4 against a background of all genes expressed during BFC differentiation (see Methods). (**C**) Distribution of Pearson product-moment correlation coefficient of expression levels of all TFs from Clusters 1 and 4, as well as controls (all TFs), respectively, with *Ucp1* expression levels in the BAT of BXD mouse reference panel strains (top) and human clonal BAT-derived cell lines (bottom). (**D**) Heatmap representing mean expression levels (row-wise z-scores) of Cluster 1 and Cluster 4 TFs and the correlation of their expression with *Ucp1* expression levels in both the mouse BXD panel and across human clonal cell lines.

Having validated our experimental system, we subsequently set out to identify novel transcriptional regulators that control the molecular processes driving BFC differentiation. To do so, we subset the transcriptome on the set of 964 significantly differentially expressed genes and observed that the individual samples clustered by replicate and according to the chronological progression along the differentiation trajectory (Fig. S1B). This suggests that the differentially expressed genes are able to explain the principal transcriptomic changes that characterize differentiating brown preadipocytes. We then used fuzzy c-means clustering (see Methods) to derive distinct gene expression patterns commonly characterizing differentially expressed genes, rather than defining simplified up- and down-regulated gene categories. Using this approach, four distinct gene expression trends, highlighting stage-specific regulators, were revealed. Genes in Cluster 1 showed increasing expression during differentiation and included key known regulators such as *Pparg, Ppara* and the brown fat marker *Ucp1* (Fig. 2A). These genes were significantly functionally enriched for gene ontology (GO) terms pertaining to BFC differentiation, cellular response to insulin, and metabolic processes characteristic of brown adipocytes, suggesting that TFs that are members of this cluster may positively regulate BFC differentiation (Fig. 2B). Notably, this cluster contained TFs that have not been characterized yet in the context of BFC differentiation, including ELF3, YBX2, NFIA, HOXA4, ZFP467, RREB1, SOX18 and DBP (Supplementary Table 1, **TF-Cluster1**).

In contrast to Cluster 1, Cluster 4 comprised genes up-regulated during the first 2 hours post induction, followed by a rapid reduction in gene expression, to levels inferior to their initial expression by Day 4 (Fig. 2A). Thus, consistent with the expression profile, Cluster 4 was more strongly enriched for functional terms characteristic of early differentiation such as cell proliferation, response to external stimulus, while functional enrichment terms pertaining to BFC differentiation were absent (Fig. 2B). TFs within this cluster that remain uncharacterized during the BFC differentiation process include factors such as ATF3, GLIS1, ETS2, SCX, CREM and SOX13 (Supplementary Table 1, **TF-Cluster4**). Additionally, we found two clusters that reflect specific temporal gene expression dynamics during BFC differentiation and that are mirror images of one another. Cluster 2 consisted of genes that are down regulated at Day 1 during differentiation and up-regulated thereafter, suggesting their possible involvement in maintaining the differentiated adipocyte stage or the requirement for their suppression at the end of the replicative adipogenic phase (Fig. S1C). In contrast, Cluster 3 comprised genes that are up regulated at Day 1 and that therefore may be required during the early stage of differentiation, again, possibly in connection with the cell cycle, but not necessarily for the maintenance of the differentiated state (Fig. S1C). While many transcriptional regulators within these two classes also remain uncharacterized with respect to their role during BFC differentiation, the less intuitive nature of their gene expression pattern and molecular function (Fig. S1C,D) led us to focus on Clusters 1 and 4 for downstream analyses.

### Putative positive transcriptional regulators induced during BFC differentiation correlate with *Ucp1* expression across multiple mouse and human datasets

In order to validate the molecular relation between the induction of the brown adipogenic phenotype and increase in the expression of Cluster 1 TFs, we explored two different, publicly available gene expression datasets, obtained from: (1) *in vivo* BAT from the mouse genetic reference panel (BXD) comprising 37 distinct genotypes^31^ (see Methods) and (2) clonal cell lines derived from human BAT^22^. We assessed the correlation between gene expression levels of each transcriptional regulator included in the four different clusters and *Ucp1* (Fig. 2C) across this large number of samples. We observed a significantly higher correlation with *Ucp1* for regulators from Cluster 1 compared to those from Clusters 4 (Fig. 2C), 2, or 3 (Fig. S1E) in both datasets. Further, upon evaluating the mean gene expression levels (across mice and across human cell lines, respectively) of each of these regulators and their correlation with *Ucp1* levels, we identified a core group of factors that exhibited both, expression in mouse and human BAT as well as a strong positive correlation with *Ucp1* levels (Fig. 2D). These included known regulators such as PPARγ, C/EBPα, PPARα, STAT5A in addition to uncharacterized ones such as RREB1, NFIA and DBP, yielding an interesting set of putative, novel regulators of brown adipogenesis (Fig. 2D).

### The active chromatin landscape is established by Day 1 of murine brown adipogenesis

Next, we set out to examine how the identified regulators may contribute to BFC differentiation. For this purpose, we evaluated active chromatin state changes during differentiation by assessing the gain or loss in acetylation on lysine 27 of the H3 histone (H3K27ac) using ChIP-seq^32^,^35^ (Fig. 1D) and subsequently explored the overrepresentation of binding sites of putative regulators within H3K27ac-enriched regions.

We first analyzed the dynamics of H3K27ac during the course of differentiation, specifically identifying regions of high H3K27ac (referred to as “acetylation”) ChIP-seq enrichment at a particular time point relative to all the others (absolute log2 FC >= 2, FDR <0.01, see Methods). We detected 2,866 differentially acetylated regions at Day 0, 2,808 regions at 2 h, 2,151 regions at Day 1, 2,205 regions at Day 2, and 2,819 regions at Day 4. We quantified H3K27ac enrichment inside the union of these dynamically acetylated regions and performed hierarchical clustering, observing that preadipocyte replicates (Day 0 and 2 h) clearly separated from differentiating/mature-specific samples (Day 1, Day 2 and Day 4) (Fig. S2A). Consistently, H3K27ac levels were largely similar at D0 and 2 h and dramatically different from those at Days 1, 2, and 4 (Fig. S2B). In contrast, regions showing strong acetylation at Day 1 were also highly enriched at Days 2 and 4 (Fig. S2B). These results support the quality of our H3K27ac data and broadly suggest that significant chromatin remodeling events take place up to Day 1 of brown adipogenic differentiation, after which the set of H3K27 acetylated regions remains relatively stable.

**Figure 3:**
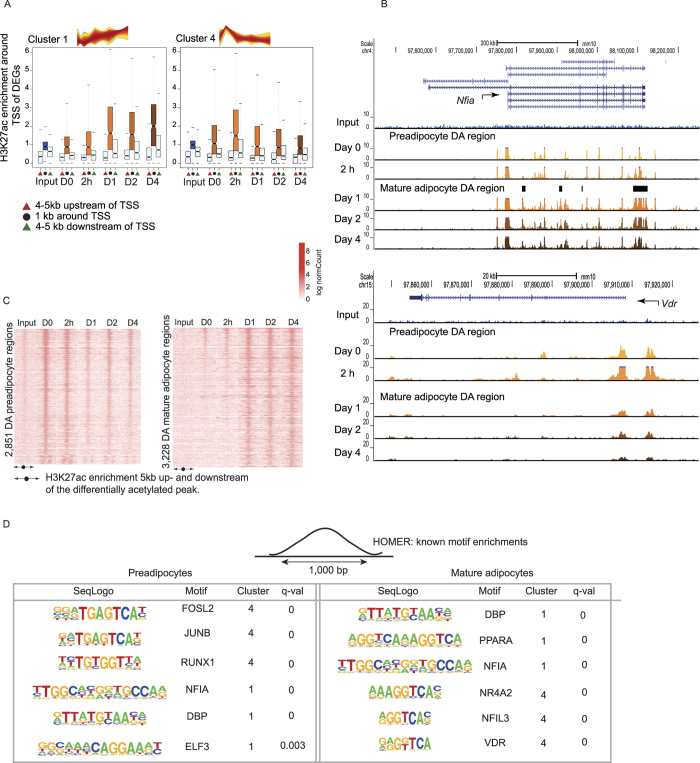
The chromatin landscape is established by Day 1 of brown adipogenesis. (**A**) The distribution of H3K27ac ChIP enrichment within a 10 kb region around the transcription start site (TSS) of differentially expressed genes (DEGs) from each cluster. The region around the TSS is partitioned into three compartments: a red triangle represents a region from +4 to +5 kb of the TSS, a black dot represents a region from +1 to −1 kb around the TSS and a green triangle represents a −4 to −5 kb of the TSS. (**B**) UCSC browser screen shots of the H3K27ac change around the genomic locus of two candidate regulators, *Nfia* (Cluster 1) and *Vdr* (Cluster 4). (**C**) Heatmap representing H3K27ac ChIP enrichment within a 10 kb region around the differentially acetylated (DA) region enriched in either Pre- or Mature- adipocytes. (**D**) Top overrepresented motifs in either Pre- or Mature adipocyte H3K27ac regions for TFs that are significantly differentially expressed across differentiation (see Methods).

To subsequently examine the correlation between gene expression and active chromatin state changes, we quantified the acetylation signal in a region 5 kb upstream and downstream of the transcription start site (TSS) of differentially expressed genes in each detected Cluster. We observed that, consistent with the positive relationship between gene expression and H3K27ac levels^32^,^35^, there is a significant increase in the H3K27 acetylation around the TSS of Cluster 1 genes during differentiation (Fig. 3A). Indeed, the median acetylation intensity around 1 kb of the TSS of differentially expressed genes in Cluster 1 in Day 0 and 2 hour samples remained significantly lower compared to the Day 1–4 samples (Wilcoxon’s paired non-parametric test, p-value <10^−16^) (Fig. 3A). While acetylation was highest at Day 4, overall, it remained relatively stable after Day 1, consistent with the chromatin landscape being set by this time point. The relative agreement between gene expression dynamics and active chromatin state changes was also observed for the other Clusters. For example, Cluster 4 genes exhibited an increase in acetylation signal at 2 h followed by a rapid decay thereafter (Fig. 3A). Representative genome browser views showing H3K27ac levels during brown adipogenesis at TF-coding genes such as *Nfia* (Cluster 1), *Vdr* (Cluster 4), *Klf4* (Cluster 2), *Tead4* (Cluster 3) as well as the marker genes *Ucp1* and *Pparg* are shown in Fig. 3B and Fig. S2D.

Given the observed similarity between Day 0 and 2 h H3K27ac profiles as well as that between Day 1, 2, and 4 profiles, we simplified our analyses, focusing on “Preadipocyte-specific regions” (enriched at Day 0) and “Mature adipocyte-specific regions” (enriched at any of Days 1, 2 and/or 4) (Fig. 3C). 2,851 regions showing significantly higher H3K27ac levels at Day 0 compared to Days 1, 2 and 4 (i.e. ~20% of all Day 0 regions) and 3,228 regions showing significantly higher H3K27ac levels at Days 1, 2, and 4 compared to Day 0 (i.e. ~10% of all the Day 1, Day 2 and Day 4 H3K27ac regions combined) were derived for downstream analyses (Fig. 3C and S2E). We note that only a minority of both these two types of regions was TSS proximal (categorized as −1kb to +100 bp around the TSS of genes) (Fig. S2F), consistent with the high dynamicity of enhancer regions across differentiation^36^. Functional enrichment analysis of the genes corresponding to these regions illustrated the overrepresentation of terms pertaining to BFC differentiation and lipid catabolism-related processes in Mature Adipocyte sites compared to Preadipocyte sites. The latter showed overrepresentation of functional terms pertaining to general biological processes relevant for initiating cellular differentiation such as actin-filament remodeling and cell morphogenesis (Fig. S2G).

In sum, we demonstrate the consistency between H3K27ac and transcriptomic changes measured at matched time points during BFC differentiation. We find that considerable chromatin remodeling occurs up to Day 1 of differentiation, after which only minor changes are observed, suggesting that the chromatin landscape of BFC is set early in the differentiation process.

### Motif enrichment at differentially acetylated regions identifies putative regulators of BFC differentiation

We next exploited the H3K27ac data to determine factors exerting a direct regulatory activity during brown adipogenesis. We reasoned that TF binding sites (TFBS) that were overrepresented in either Pre- or Mature-adipocyte-specific H3K27ac enriched regions would be indicative of directly involved TFs. We thus took an unbiased approach, retrieving known mouse TF DNA binding motifs from the recently updated HOCOMOCO database^37^, which contains over 700 position weight matrices (PWMs). We performed differential motif discovery in preadipocyte and mature adipocyte H3K27ac-enriched regions including both promoter- proximal and -distal regions, using HOMER (see Methods).

This analysis revealed substantial differences between early and late active regions, broadly suggesting that different transcriptional regulators operate at distinct stages of BFC differentiation. We identified 215 motifs enriched in Preadipocyte regions and 132 enriched in Mature adipocyte ones (q-value cutoff of 0.01, see Methods and Supplementary Table 2). Preadipocyte regions were enriched in (1) motifs of known early response TFs, including JUN, AP1, FOS; (2) ATF3, known to play a role in adipocyte hypoxia-mediated mitochondrial dysfunction in obesity^38^; (3) a set of TFs whose role in brown adipogenesis has not yet been established such as RUNX1, DBP, ELF3, and NFIA (Fig. 3D, Supplementary Table 2 and 3). As expected, we observed that Mature adipocyte-specific acetylated regions enriched for motifs of known and well characterized adipogenic regulators such as C/EBPs, PPARγ, PPARα, as well as members of the hormone nuclear receptor family such as NR4A2 and NFIL3 (Fig. 3D, Supplementary Table 2 and 3). In addition, these regions were enriched for motifs of TFs that have so far not been implicated in brown adipogenesis such as DBP, implicated in controlling the adipocyte clock^39^, and again NFIA, implicated as a positive regulator of white adipocyte differentiation^40^ (Fig. 3D, Supplementary Table 2 and 3). We note that more generally, several of the TFs with overrepresented motifs are known regulators of white adipogenesis, whose molecular involvement in BFC differentiation has not been established yet (NFIA, DBP).

In sum, by delineating Pre- versus Mature adipocyte-specific H3K27ac-enriched regions, we were able to uncover several putative, novel BFC differentiation regulators. Particularly, motifs of TFs from Cluster 1 such as NFIA and DBP were enriched in both acetylated region groups whereas the ELF3 motif was only detected in Pre-adipocyte regions. A majority of these TFs also showed dynamic gene expression levels during BFC differentiation, most notably high correlation with *Ucp1* expression in the case of NFIA, strongly supporting their direct regulatory involvement in brown adipogenesis.

### Loss of function assays identify novel regulators impacting brown adipogenesis

Combining our results from transcriptomic, epigenomic, and differential motif discovery analyses, we produced the following set of putative, novel positive regulators of BFC differentiation: NFIA, DBP, HOXA4, ZFP467, SOX18, ELF3, YBX2, (all belonging to Cluster 1), along with the “positive control”, PPARα^41^. To assess the importance of each of these regulators during the course of BFC differentiation, we perturbed their gene expression levels via lentivirus-mediated shRNA knockdown (KD) using at least two different short hairpin RNAs per gene (see Methods). To analyze the regulatory effects of these KDs at a systems level, we profiled their transcriptomic outcomes using a novel multiplex, bulk mRNA-seq barcoding method (see Methods). Using this approach, we sequenced the 3′ ends of mRNAs obtained from each individual KD and compared it with three types of controls: cells not transduced by viruses (Non transduced control), cells transduced with a scrambled short hairpin RNA construct (Scrambled control) and cells transduced with a GFP containing plasmid (GFP control). The underlying goal was to examine how the transcriptome of the perturbed TFs deviated from the control samples in mature adipocytes. We first validated our sequencing approach by correlating the estimated *Ucp1* expression levels with those obtained by qPCR. We observed a strong positive correlation (Pearson’s correlation coefficient of 0.99) between the two measurements, demonstrating the accuracy of bulk 3’ end mRNA-seq expression data (Fig. S3A).

**Figure 4:**
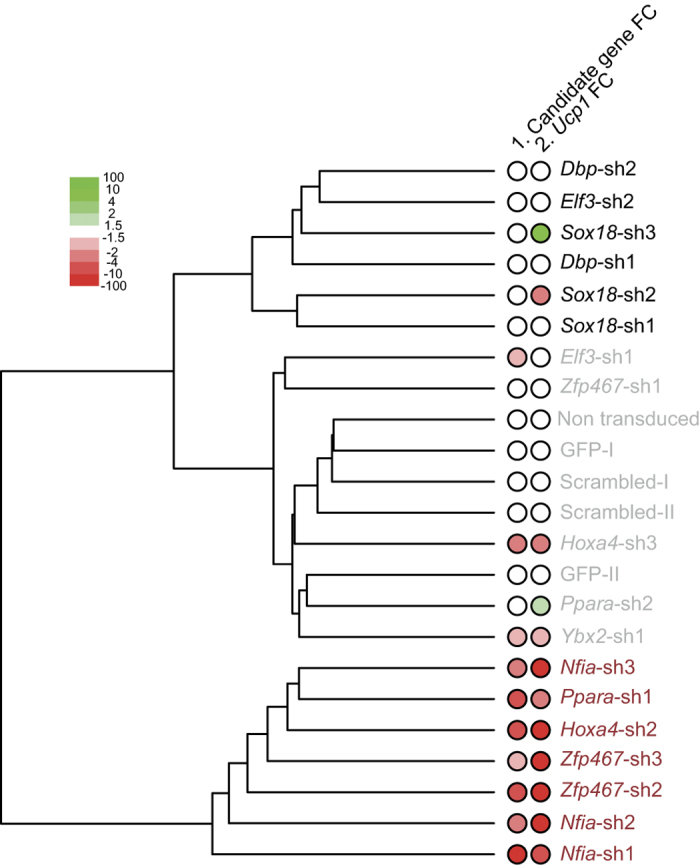
Transcriptomic and phenotypic outcome of candidate TF perturbation. Hierarchical clustering of controls and knockdowns (KDs) performed with distinct shRNA constructs. Column 1 indicates the degree of TF KD and Column 2 indicates the reduction in *Ucp1* expression levels.

To achieve a comprehensive overview of transcriptomic perturbations upon KD of the distinct putative regulators, we first clustered the samples based on their complete transcriptome (Fig. 4). To determine if the observed transcriptome perturbations (if any) were caused by actual depletion of the respective regulators, we also plotted the KD efficiencies as well as noted changes in *Ucp1* expression (Fig. 4). This analysis led to the identification of three principal clusters guided by their transcriptomic variability (Fig. 4): cluster I comprised *Zfp467, Hoxa4* and *Nfia* (expression Cluster 1) whose regulatory function during BFC differentiation has not been established yet, next to the known regulator *Ppara*; cluster II comprised controls along with known and putative positive regulators such as *Ybx2, Elf3* (expression Cluster 1) and *Ppara* (known positive regulator of BFC and expression Cluster 1); cluster III comprised another set of putative positive regulators such as *Sox18, Elf3* and *Dbp* (expression Cluster 1), which grouped apart from cluster I due to discrepancies in KD efficiencies and technical variability (see Methods). The samples in the first cluster showed a strong KD and impact on the brown adipocyte phenotype given the significant decrease in *Ucp1* expression. The anomalous cluster III consisted of factors that had either not been sufficiently knocked down or the estimation of the number of reads was poor due to low sequence coverage. Further, certain discrepancies existed for factors such as *Sox18* sh2 and sh3 for which *Ucp1* levels fluctuated, even though *Sox18* itself had not been knocked down, owing to low sequence coverage. In sum, upon evaluating the factors based solely on their effect on the efficiency of KD and impact on *Ucp1* expression, the cluster I factors *Nfia Hoxa4*, and *Zfp467* emerged as the most promising novel candidate regulators of brown adipogenesis.

### NFIA is a key early regulator of murine terminal BFC differentiation.

**Figure 5:**
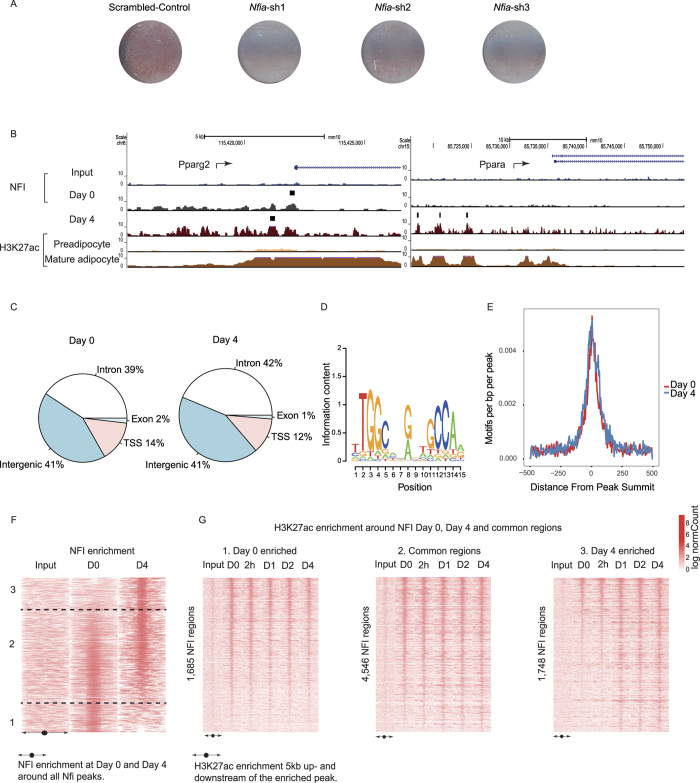
NFI binding is associated with active chromatin near key BFC marker genes. (**A**) Lipid accumulation visualised using Oil red O (ORO) staining of scrambled control versus *Nfia* KD samples. (**B**) UCSC browser screen shots of NFI binding sites at the *Pparg2* promoter and distal to the *Ppara* promoter including H3K27ac ChIP-seq coverage profiles associated with Pre- and Mature- brown adipocytes. (**C**) Overview of NFI bound sites at their genomic locations at Day 0 and Day 4. (**D**) NFIA PWM derived from HOCOMOCO and used for scanning NFI peaks. (**E**) NFIA motif localization at Day 0 and Day 4, in a 1 kb region around the summit of NFI peaks. (**F**) NFI enrichment at Day 0 and Day 4 around 7,979 NFI bound regions across Day 0 and Day 4. (**G**) H3K27ac ChIP-seq enrichment within a 10 kb region surrounding NFI peaks enriched at Day 0 (group 1, 1,685 regions), common to both Day 0 and Day 4 (group 2, 4,546 regions) and enriched at Day 4 (group 3, 1,748 regions).

Among candidates identified by our integrative approach, NFIA was the strongest candidate given (1) its correlation with increasing *Ucp1* levels during differentiation, (2) association with mean *Ucp1* expression levels in the BXD reference panel and human BAT clonal cell lines, and (3) overrepresentation of its motif in both Pre- and Mature adipocyte-specific active chromatin regions. Visualisation of lipid accumulation upon *Nfia* KD revealed a strong phenotypic impact on the process of differentiation (Fig. 5A), dependent on the efficiency of the KD (Fig. S3B). Given that the impact of *Nfia* perturbation on brown adipogenesis had not been described previously, we chose to study its regulatory function in more detail by identifying its genome-wide target sites. Due to the lack of a murine, NFIA-specific antibody, we used a generic NFI (Nuclear Factor 1) antibody for ChIP-seq at two different time points, Day 0 (Preadipocyte) and Day 4 (Mature adipocyte).

We observed that NFI occupies genomic positions around the TSS of established adipogenic regulators such as *Pparg2* and *Ppara* (Fig. 5B). Of the 4,375 and 4,473 NFI-bound regions detected at Day 0, and 4 respectively, the majority was either intronic or intergenic, albeit still in close proximity to genes (Fig. 5C). To validate our ChIP-seq assay, we focused on specifically determining the presence of the NFIA DNA-binding motif within peaks (Fig. 5D), observing its central enrichment around NFIA peak summits at Day 0 and Day 4 (Fig. 5E). Interestingly, when assessing H3K27ac enrichment around NFI-enriched peaks, we observed a strong association with H3K27ac enrichment across BFC differentiation. Specifically, Day 0- and Day 4-specific NFI peaks (Fig. 5F, see Methods) showed greatest H3K27ac enrichment at these same, respective time points, whereas NFI peaks that were common to both Days 0 and 4 exhibited similar H3K27ac enrichment throughout all time points (Fig. 5G). These data point to a clear relationship between active chromatin and NFI binding (Fig. 5G).

**Figure 6:**
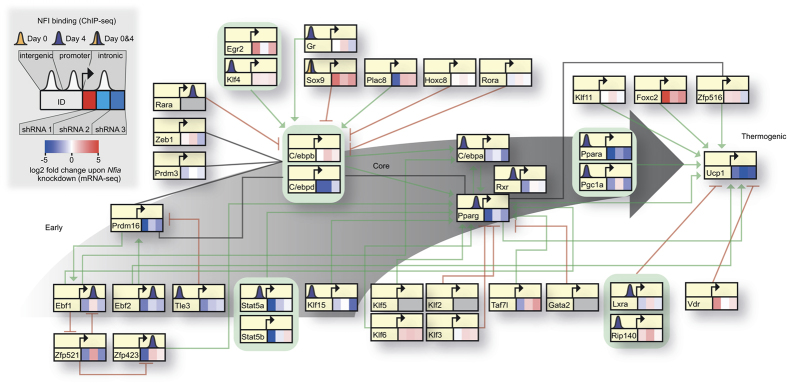
NFIA as a key early regulator of terminal brown fat cell differentiation. Summary of the adipogenic and brown adipogenic gene regulatory network (GRN) and the transcriptomic impact upon *Nfia* perturbation using shRNA mediated loss of function assays. Approximate binding locations (ChIP-seq based) of NFI are represented. Green arrows represent activation, red t-bars represent inhibition while grey thick lines represent protein-protein interactions. Each node is divided into three boxes representing the gene expression levels using three different shRNAs. Additionally, a small peak colored either yellow or purple represents NFI binding either at Day 0 or Day 4, with the peaks distributed according to their spatial location: promoter, intergenic or intronic.

Next, we examined if NFI ChIP-seq peaks were located near adipogenic or BFC-specific genes. To address this, we generated a comprehensive brown fat gene regulatory network (reviewed in ref. 6), including early, core and thermogenic regulators, and probed the effect that perturbation of NFIA has on this network, visualizing both gene expression changes induced by *Nfia* KD (per shRNA) on the expression level of each node (Fig. 6 and S3B)and NFI genomic occupancy as measured by ChIP-seq (Fig. 6). This revealed that *Nfia* KD severely impaired *Pparg* expression as well as the brown phenotype by negatively affecting *Ucp1* expression. However, *Ucp1* does not seem to be directly targeted by NFIA. Instead, NFIA appears to regulate this process by binding to the promoters of (1) the core adipogenic regulators *Pparg* and *Rxrg*, (2) the drivers of the thermogenic module such as *Ppara* and *Pgc1a*, as well as (3) the early adipogenic circuit regulators *Ebf1* and *Ebf2*, as well as *Zfp423* (Fig. 6). It has been previously demonstrated that Zfp423 is required in the initial formation of both brown and white adipocytes *in vivo*, suggesting a molecular role of Zfp423 in adipogenic commitment. Our observation of NFIA binding to *Ebf2* and *Zfp423* is in this regard intuitive, since it suggests a dual role of NFIA in regulating early and late factors that contribute to brown adipocyte differentiation. The binding profile is in coherence with changes in gene expression observed for each of these factors upon *Nfia* KD. Additionally, we sought to probe the co-localization of TFBS along with NFIA that may work in concert with NFIA to facilitate its pro-adipogenic activity (see Methods). This analysis revealed a distinct set of factors in differentially acetylated pre- and mature adipocyte sites containing an NFI peak (Fig. S3C). In preadipocytes, NFIA is predicted to occur along with (1) known early response TFs such as JUN and FOS, (2) TFs from Cluster 4, ATF3 and BATF Fig. 2A, Fig. S3C and Supplementary Table 1) and (3) a TF from Cluster 3, TEAD4 (Fig. S1C and S3C and Supplementary Table 1), that are required for the early response with reduced gene expression after 2 hours and Day 1 post differentiation, respectively. While ATF3 is implicated in mitochondrial dysfunction in obesity^38^, BATF and TEAD4 are completely uncharacterized in the context of adipogenesis. Most notably, in mature adipocytes, NFIA is predicted to occur along with known key molecular players of adipogenesis such as PPARγ, EBF, C/EBPβ, RXR and C/EBPα. This suggests the communal action of these TFs along with NFIA to execute pro-adipogenic gene expression programs (Fig. S3C). Overall, these data strongly suggest that NFIA acts a key direct regulator of terminal BFC differentiation, propagating its regulatory effect on *Ucp1* via the early, core and thermogenic circuits.

## Discussion

In this study, we performed to our knowledge the first comprehensive analysis of the transcriptomic and epigenomic changes during brown adipogenesis. These experiments were performed using an *in vitro* differentiation system, given the intrinsic difficulty of studying BFC differentiation *in vivo*. While obviously limited in its capacity to mimic the *in vivo* process, we showed here that this system produces brown fat cells that are molecularly and phenotypically comparable to their *in vivo* counterparts. As such, a conceptual parallel can be drawn with the 3T3-L1 cell line, which proved key in uncovering the regulatory network that controls white adipogenesis^7^,^10^,^11^,^12^,^13^,^14^,^15^,^16^,^17^. Similarly, the genomic data generated in this study should constitute an important resource to study the regulatory mechanisms underlying BFC differentiation.

We observed that the transcriptomic changes characterizing the BFC differentiation process could be classified into four temporally distinct gene expression clusters, followed among others by several transcriptional regulators not previously implicated in brown adipogenesis. Consistent with the well-established notion of gene expression maintenance through epigenomic modifications, regulated by TFs and chromatin regulators^35^, we observed coherence between gene expression patterns and the chromatin state, defined by H3K27ac enrichment. For example, during differentiation, we observed an increase in H3K27ac levels around the TSSs of Cluster 1 genes such as *Ucp1* and *Pparg*, in line with the high H3K27ac enrichment around these genes previously reported in whole BAT^42^. In contrast, genes from Cluster 4 such as *Vdr* showed a decrease in H3K27ac levels, consistent with their decreasing gene expression along differentiation (Fig. 3C) as well as with previous studies demonstrating the negative differentiation effects of ATF3, IRX3, IRX5, NR4A1 (NUR77) (expression Cluster 4) on the metabolic function of brown fat^43^,^44^,^45^,^46^. Globally, more H3K27ac regions are gained from 2 hours to Day 1 (1,322) than from Day 1 to Day 2 (15) and Day 2 to Day 4 (205) (Fig. S2B), clearly suggesting the establishment of the acetylation landscape within the first day post addition of the induction cocktail. These observations parallel those from open chromatin profiling analyses during white adipogenesis, which showed that the chromatin landscape is established prior to Day 1 post differentiation induction^17^. Interestingly, this time window coincides with the mitotic clonal expansion phase, characteristic of *in vitro* adipocyte differentiation^47^, suggesting an intricate relationship between chromatin remodeling and DNA-template based processes such as the cell cycle^48^.

Given the demonstration of the feasibility of identifying putative, novel transcriptional regulators mediating white adipogenic differentiation using H3K27ac-enriched regions^16^, we used a similar approach to detect overrepresented TFBSs of TFs identified through transcriptomic profiling within Preadipocyte and Mature adipocyte-specific H3K27ac-enriched regions. Through integrative genomics, we identified several putative novel transcriptional regulators of BFC differentiation such as NFIA, HOXA4, ZFP467, DBP, ELF3, and SOX18. Among those, NFIA, ZFP467 and HOXA4 have already been associated with white adipogenesis^40^,^49^,^50^,^51^, suggesting that the regulatory mechanisms underlying the core differentiation program are shared between white and brown adipogenesis. In contrast, none of the other TFs has to our knowledge so far been studied in an adipogenesis context, making them interesting candidates for follow-up studies. After testing the transcriptomic and phenotypic alterations in response to KD of these putative regulators, NFIA emerged as the factor showing strongest effects. Its correlation with *Ucp1* expression in our *in vitro* differentiating cell line, in BAT from the mouse BXD panel, and in clonal human BAT derived cell lines lends further support to the importance of NFIA in BFC differentiation.

NFIA is a member of the well-known NFI (Nuclear factor I) family of TFs, including members such as NFIB, NFIC and NFIX. Each of these TFs are known to be widely expressed, with different mouse knockout models yielding distinct phenotypes, suggesting that each NFI TF regulates a distinct set of genes^52^. We found *Nfia* to be significantly up regulated during the course of BFC differentiation in our *in vitro* system, suggesting that it may regulate a set of adipogenic and/or brown adipogenic genes. In addition, we found the NFIA motif to be enriched in active chromatin regions, which could be confounded by motif closeness to other family members, given that NFI factors tend to bind to similar dyad–symmetric TTGC(N)_5_GCAA sequence motifs^53^,^54^. Interestingly, NFIA has been implicated as a positive regulator in adipogenesis, specifically in white adipocyte differentiation, based on results in 3T3-L1 cells^40^. The study clearly demonstrated that adipogenic genes such as *Cebpa, Pparg2* and *Ap2*, are activated through direct binding of NFI to their regulatory elements. These findings strongly align with our observations regarding NFI binding to *Pparg* and *Cebpa* as well as to brown-specific regulators such as *Ppara* and *Pgc1a*. Our analyses implicate NFIA in brown adipogenesis along with its previously established role as a positive regulator of white adipogenesis^40^. Given that we are limited by the lack of an NFIA-specific antibody, our observations represent an approximation of the localization of this factor. Nevertheless, perturbation analyses strongly support our claim of NFIA being a strong positive regulator capable of controlling the brown adipogenic program via propagation of its effect through the early, core and thermogenic modules (Fig. 6). We speculate that NFIA may exert its regulatory function by making important enhancer or promoter elements accessible to other brown adipogenic TFs based on the strong correlation between NFI binding and H3K27ac enrichment both in Pre- and Mature adipocytes (Fig. 5F,G). Such a hypothesis is consistent with previous findings, revealing a role for NFI in chromatin remodeling as well as association with active chromatin^54^,^55^. Future work will need to focus on exploring this hypothesis and elucidating the mechanistic details of NFIA action during BFC formation and activation.

Our transcriptomic analyses of *in vitro* BFC differentiation highlighted a panel of transcriptional regulators that strongly correlated with the brown adipogenic molecular phenotype. Importantly, we have shown that for several factors, this molecular relationship holds true *in vivo*, across tens of distinct genotypes, as assessed by expression data from the BAT of BXD mice^31^, and even translates to humans, as demonstrated in clonal BAT-derived cell lines (Fig. 2D). Moreover, we took a highly multiplexed barcoded mRNA-seq approach to obtain a comprehensive snapshot of the global transcriptomic impact of knocking down these TFs of interest and thus an unbiased first glance into their mechanism of action. Our combined analytical and phenotypic results revealed for the first time that the TFs NFIA, HOXA4 and ZFP467, are potentially important positive regulators of brown adipogenesis. Some of these TFs (NFIA, HOXA4, and ZFP467) have been previously implicated in the general adipogenic regulatory program, making their involvement in the BFC regulation reminiscent of the molecular function of PPARγ, required for both differentiation processes. However, unlike PPARγ, their mechanism of action has only been marginally studied and thus represents putative fruitful avenues towards the ultimate goal of efficient and safe organismal-level energy modulation. We envision that our study will contribute to the expansion of the current brown adipogenic gene regulatory network in addition to providing a framework for the analysis of other differentiation-based processes.

## Materials and Methods

### Cell culture

The immortalized murine-derived brown pre-adipocyte cell line (IBA) was derived from the stromal vascular fraction (SVF) of interscapular brown adipose tissue of young male (C57BL6/J)^20^ provided by Prof. Christian Wolfrum’s laboratory (ETH-Zurich) and prepared according to standard protocols. IBA was cultured in high-glucose Dulbecco’s modified Eagle’s medium (DMEM, Life Technologies) supplemented with 10% fetal calf serum (FCS, AMIMED), with 100X penicillin/streptomycin/glutamine (100x Pen/Strep/Glutamine, Life Technologies) in a 5% CO_2_ humidified atmosphere at 37 °C and maintained at less than 80% confluence before passaging. Differentiation of IBAs was induced by exposing confluent cells (Day 0) to DMEM containing 10% FCS supplemented with 0.5 mM 3-isobutyl-1-methylxanthine, 1 uM dexamethasone, 1.96 nM insulin (Sigma, stock solution of 167uM prepared in PBS), 125 uM indomethacin (Sigma, stock solution of 18 mM prepared in absolute ethanol), 1 nM T3 (3,3′,5-Triiodo-L-thyronine sodium salt, Sigma, prepared in 45 mM KOH). After exactly 28 hours, the induction medium was replaced with adipocyte differentiation medium consisting of 1 nM T3 and 1.96 nM insulin. Cells were maintained in this medium until Day 5 and were stained with Oil Red O for lipid accumulation at Day 6 as full differentiation is achieved by then.

### Staining

Cells were washed twice with PBS and fixed in 4% paraformaldehyde (PFA) in PBS solution for 15 minutes. After 2 washes in PBS, the samples were permeabilized for 5 minutes with 0.5% NP40 in PBS. Next, the samples were washed twice in PBS-TX (PBS +0.01% Triton-X100). The samples were blocked for 1 hour in blocking buffer (PBS +3% BSA (w/v) +0.05% Triton-X100) at room temperature (RT). Next, the TOM20 primary antibody (Santa Cruz) (1:500 in blocking buffer) was incubated overnight at 4 °C. After three washes with PBS-TX, the samples were incubated with an Alexa568 labeled secondary antibody (1:1000 in blocking buffer) for 1 hour at RT. The sample was washed once with PBS and subsequently incubated for 5 minutes at RT with PBS containing 1:500 BODIPY and 1:2000 DAPI. Finally, the samples were washed twice with PBS and imaged in PBS on a Zeiss LSM700 invert confocal microscope. These images were processed by adapting the brightness and contrast of the DAPI channel using ImageJ. Lipid accumulation was determined using Oil Red O stain at Day 6 of differentiation as previously described^10^.

### Loss of function assay

Viral particles containing shRNA expression plasmids (Supplementary Table 4) were generated in 293 T cells as described previously^56^, with slight modifications. Instead of five plates, viruses were packed in 6 well plates and harvested once after 48 hours post transfection of plasmids. The supernatant was collected and the cell debris removed by centrifugation. Transient knockdown assays were performed wherein the cells were incubated with viral supernatant in a ratio 1:3 (complete medium: viral supernatant), at confluence. Medium was refreshed after 24 hours and differentiation was induced 24 hours after the medium change. Transcriptomic and phenotypic readouts were obtained at Day 6 of differentiation (Figs 4 and 5A).

### RNA isolation and quantitative PCR

Total RNA was extracted using a Qiagen RNAeasy plus mini kit/Zymo Direct-zol kit according to manufacturer’s protocol by using either the RLT buffer lysis system or the TRIzol/Chloroform extraction procedure (Sigma) respectively. The RNA concentration and quality was determined using a nanodrop (1.8 ≤ A260/A280 ≤ 2.2) and by visual inspection of separated bands on Fragment Analyzer (Advanced Analytical). 1.5–2 μg (depends on RNA concentration) of total RNA was used for single strand cDNA synthesis using the SuperScript VILO cDNA Synthesis kit (Life Technologies). Then, cDNA was diluted 1:10 using nuclease free water and 1.5 μl was used for each qPCR reaction. Quantitative real-time PCR was performed in 384 well plates with three technical replicates on the ABI-7900HT Real-Time PCR System (Applied Biosystems) using the Power SYBR Green Master Mix (Applied Biosystems) using standard procedures. A Hamilton Liquid Handling Robotic system was used to assemble the 384 well plates. The qPCR primers (available upon request) were designed with in-house developed GETPrime software^57^ or taken from previous publications. They were checked for linearity and single product amplification.

### Western Blot analysis

Protein expression measurements were conducted using cell lysates from maturing brown adipocytes at Day 0, Day 4, Day 6 (with and without isoproterenol treatment). Cell lysates were prepared in 1.2X Laemelli buffer, sonicated at high speed, 30 s ON/OFF cycles, for 10 minutes and denatured at 95 °C for 5 minutes. Standard SDS–PAGE was performed using 10–12% resolving gels and transferred to a nitrocellulose membrane for probing. The following proteins were probed (1) UCP1 (using Abcam anti-UCP1 antibody) (2) PPARγ (using Santa Cruz anti-PPARγ antibody) (3) α-TUBULIN (using Sigma anti- α-TUBULIN antibody).

### mRNA sequencing

After isolation of RNA, as described in the section **“RNA isolation and quantitative PCR”**, the Illumina Truseq RNA sample preparation kit v2 protocol (Illumina, USA) was followed using 500 ng of RNA per sample as starting material. Libraries were checked for quality and quantified using the Bioanalyzer 2100 (Agilent, USA), before being sequenced (single end sequencing at UNIL; and GECF, EPFL). Two libraries (technical replicates 1 and 2) were prepared according to the un-stranded Illumina Truseq RNA sample preparation kit v2 protocol while the third biological replicate was prepared using the stranded protocol. This dataset has been deposited in ArrayExpress, currently awaiting the provision of an accession number.

### mRNA-seq computational analysis and detection of differentially expressed genes

Sequenced tags were aligned to the Ensembl 75 gene annotation of the NCBI38/mm10 genome using Tophat2 (Version 2.0.11), and the parameters “-p 8 -b2-sensitive” were used for unstranded libraries and, “-library-type fr-firststrand” was used for stranded libraries. Expression levels per gene (counts over exons) were estimated using HTseq (Version 0.6.1)^58^ using the parameters “htseq-count -f bam -s no -t exon -i gene_id -m union” for un-stranded and “htseq-count -f bam -s reverse -t exon -i gene_id -m union” for stranded libraries. All downstream analyses were performed using R (Ver 3.0.2)^59^. Genes with FPKM <1 at any of the time points during differentiation were removed and the “expressed” transcriptome was used for downstream analyses. Library normalization was performed using edgeR (Version 3.8.6)^60^ followed by batch effect correction using ComBat (Version 3.12.0)^61^. Expression differences between samples were quantified using the limma-voom pipeline (limma Version 3.22.7)^62^,^63^,^64^, with a stringent cutoff of log2(FC) >= 2, padj <= 0.01. Hierarchical clustering of samples with all expressed genes versus differentially expressed genes was performed using Pvclust (Version 2.0)^65^, distance: correlation, cluster method: complete, with 10,000 iterations.

### Correspondence Analysis

Using correspondence analysis, we compared our *in vitro* mRNA-seq data (expressed transcriptome; voom normalized counts) with the microarray data set from *ex vivo* differentiated cells derived from different fat depots in mice (GSE39562^21^,). Specifically, we used the made4 package (Version 1.40.0)^66^ to observe data samples (cell lines/differentiating cells) and data points (genes) in one low dimensional space. We visualized this information as a biplot where each axis of the diagram revealed a profound characterization of the data set. Samples and data points having high similarity with respect to this characterization have similar coordinates on it. Plot of axis 1 against axis 2 explains technical differences between the two datasets. We then plotted axis 2 and axis 3 to capture differences that drive the separation between white and brown samples.

### Fuzzy c-means clustering

We derived gene expression trends from differentially expressed genes using a fuzzy c-means approach applied via the Mfuzz package (Version 2.26.0)^67^. We used a fuzzifier (m = 2.057216) to partition the data using a relation proposed by Schwaemmle and Jensen^68^. Cluster number was optimized by testing a range of cluster numbers with four final clusters chosen on the basis of: (1) Dmin (minimum distance between cluster centroids as a cluster validity index) (2) Increasing fuzzifier (3) Functional enrichment analysis of individual clusters to determine biological relevance. We observed that upon increasing the fuzziness of the data (i.e by increasing the partition coefficient m to 3), the genes *Ucp1* and *Pparg* switch clusters and show higher membership values to the “switched” cluster leading to the selection of four clusters. Functional enrichment analysis was performed on genes from each cluster versus all expressed genes during brown adipogenesis using topGO (Version 2.18.0)^69^ and a custom script, using Fisher’s exact test, with a p-value cutoff <0.01.

### Public dataset analysis

Mouse *in vivo* brown fat expression data was available from http://genenetwork.org/webqtl/main.py, dataset “EPFL/LISP BXD Brown Adipose Affy Mouse Gene 2.0 ST Gene Level (Oct13) RMA”. We calculated the Pearson product-moment correlation coefficient between the normalized expression level of *Ucp1* and that of all transcription factors annotated as part of Clusters 1–4 (at probe level). Values were displayed in R using the beanplot() function (package beanplot) with default parameters. As control group, we used all transcription factors expressed in the BXD dataset. Similarly, we downloaded publicly available data on clonal human BAT-derived cell lines from GEO, http://www.ncbi.nlm.nih.gov/geo/, GSE68544 and correlated *Ucp1* levels with normalized gene expression levels for all transcription factors annotated as part of Clusters 1–4 (at probe level) as well as all transcription factors expressed in the human clonal dataset, as control. Additionally, we displayed row-normalized (z-scores) mean (averaged over multiple probes, if the case) gene expression levels as well as row-normalized (z-scores) mean (averaged over multiple probes, if the case) Pearson product-moment correlation coefficients with *Ucp1* levels using heatmap.2 for all transcription factors having shown significant (multiple testing adjusted p-value <= 0.05) positive or negative correlation to *Ucp1* in mouse.

### ChIP-seq

Murine brown pre-adipocytes were collected at Day 0, 2 hours, Day 1, Day 2 and Day 4. The cells were fixed as described previously^70^ and stored at −80 °C. Ten million cells were used for each IP. The ChIP experiment was performed using magnetic beads protocol (Dynabeads) (H3K27ac and NFI). Antibodies used for each time point are: H3K27ac (Active Motif), NFI (Santa Cruz). The DNA was stored at −20 °C until the verification of ChIP enrichment by qPCR and ChIP-seq library preparation. Multiplexed libraries were prepared using barcoded adapters for each sample following the protocol described in^70^ with slight modifications. In brief, ChIP-DNA fragments (5–10ng) were end-repaired using an End-IT DNA end repair kit (Epicentre Technologies) followed by addition of an A-base and ligation of bar-coded adapters. After ligation incubation, DNA was cleaned up using AMPure XP beads (Agencourt). Two biological replicates for five time points (Day 0, 2 h, Day 1, Day 2 and Day 4) were prepared for probing H3K27ac levels during differentiation, while one biological replicate at Day 0 and Day 4 was prepared for NFI. This dataset has been deposited in ArrayExpress, currently awaiting the provision of an accession number.

### ChIP-seq computational analysis of the H3K27ac data and detection of differentially modified regions

Sequenced ChIP tags were aligned to the NCBI38/mm10 genome with Bowtie2 (Version 2.2.1)^71^ and the parameters “-very-sensitive -p 8”. Duplicates were removed and mapped reads were retained. Coverage profiles visualized using the UCSC genome browser were generated using HOMER’s makeUCSCfile.pl script for each H3K27ac replicate as well as for merged replicates at matched time points. For each replicate and time point, peaks (regions) were detected using HOMER’s (Version 4.5)^72^ findPeaks script with the parameters “region -size 1000 -minDist 2500”. Overlapping peaks between each replicate at matched time points were obtained (Day 0: 14,106, 2 h: 13,949, Day 1: 13,482, Day 2: 13,225, Day 4: 13,583) and H3K27ac tag counts were quantified using a custom script. Specifically, a union dataset of all overlapping peaks at different time points was constructed and H3K27ac tags overlapping these peaks were quantified for every time point and replicate using the countOverlaps function from the GenomicRanges package. The dataset obtained was processed exactly with the pipeline described in “**mRNA-seq computational analysis and detection of differentially expressed genes”** to derive differentially acetylated regions (with a stringent cutoff of log2(FC) >= 2 and padj <= 0.01) for downstream analyses. Time point enriched differentially acetylated (DA) regions (Fig. S2B) were derived by sub-setting on regions specifically enriched at one time point, for instance Day 0 relative to all other time points. More specifically, time series based inspection was performed to assess the gain in DA regions, leading to a gain in 220 regions from Day 0 to 2 h, 15 regions from Day 1 to Day 2 and 205 regions from Day 2 to Day 4. Preadipocyte-specific differentially acetylated regions were derived by sub-setting on regions enriched in Day 0 relative to Day 1, Day 2 and Day 4 (collectively grouped as mature adipocyte regions), while Mature adipocyte-specific DA regions were derived relative to Day 0 only. Acetylation enrichment of merged replicates per time point, within a 10 kb window of the DA site for each of the derived regions was estimated using HOMER’s annotatePeaks.pl script with the parameters “-size 10000 -hist 25 –ghist” and visualized using the pheatmap package in R (Version 1.0.2). Additionally, as a means to verify the gene expression profiles derived using the Mfuzz package, we plotted the distribution of the acetylation signal within a 10 kb window of the TSS of differentially expressed genes for each cluster. We used Wilcoxon’s paired non-parametric test to identify significant differences between Day 0 and Day 1, 2, 4 samples.

### Motif discovery within differentially acetylated regions

Motif discovery was performed using HOMER’s findMotifsGenome.pl script, within regions of size 1 kb using parameters “-size 1000 -S 25 -len 8,10,12 -p 8” and an updated set of ~700 Position Weight Matrices (PWMs) from the latest HOCOMOCO release compatible with the HOMER motif format (see Supplementary Table 2 for a complete list of motifs derived in Pre- and Mature adipocyte specific regions). HOMER generated background sequences were used for computing motif enrichment.

### ChIP-seq computational analysis of NFI data

Alignment was performed using the pipeline described in “**ChIP-seq computational analysis of H3K27ac data”.** Peaks were called using HOMER’s findPeaks.pl script using the parameters “-center -size 200”. Motif localization analysis was performed using HOMER’s annotatePeaks.pl with default parameters (“-size 1000 –hist 5 –m”). NFI enrichment at Days 0 and 4 was estimated using HOMER’s annotatePeaks.pl with the parameters “size 1000 -hist 25”, while H3K27ac enrichment was estimated with the parameters “-size 10000 -hist 25 -ghist” at the following set of sites: (1) Day 0 enriched (2) NFI regions at both Day 0 and Day 4 (3) Day 4 enriched sites. To facilitate the identification of motifs that co-localise with NFIA, we intersected NFI peaks enriched at Day 0 and Day 4 with differentially acetylated regions in preadipocytes and mature adipocytes, respectively. Motif analysis was performed as described in “**Motif discovery within differentially acetylated regions**”.

### Annotation and Functional Enrichment Analysis

H3K27ac regions and NFI bound sites were assigned to genes using HOMER’s annotatePeaks.pl function using default parameters to the mm10 genome. A region/peak is assigned as promoter proximal if it lies within a region −1kb to +100 bp of the TSS of a gene. Functional enrichment analysis was performed using topGO and a custom script as described above.

### BAT ENCODE data analysis

Additionally, we used the following publicly available data sets (1) mRNA-seq data from BAT (http://www.ncbi.nlm.nih.gov/geo/query/acc.cgi?acc=GSM929703) (2) H3K27ac ChIP-seq from BAT (http://www.ncbi.nlm.nih.gov/geo/query/acc.cgi?acc=GSM1000075). The data was processed analogous to the lab-generated data described in previous sections.

### Barcoded mRNA-seq

The method describes a time, labor and cost efficient preparation of single Nextera compatible cDNA library bearing up to 96 (and potentially more) different RNA samples (Alpern, Gardeux *et al*., in preparation). The protocol is adopted from SCRB-seq, developed for single cell transcription profiling^73^, with several important modifications. For the current series of experiments, 50 ng of total RNA from each sample was reverse transcribed in a 96-well plate using Maxima H Minus RT (Thermo) with individual oligo-dT primers, featuring a 6 nt long multiplexing barcode, and template switch oligo (Microsynth). Specifically, each oligo-dT primer is biotinylated and has the following structure: 5′-ACACTCTTTCCCTACACGACGCTCTTCCGATCT[BC_6_] [N_15_] [T_30_] VN-3′, where, [BC_6_] = 6 nt barcode specific to each well; N_15_ stretch of random nucleotides forming a Unique Molecular Identifier (UMI). Thus, for each well we have generated a unique combination of barcodes and UMIs to identify each well (sample) and transcript. Next, all the samples were pooled together, purified using DNA Clean and Concentration kit (Zymo), and treated with exonuclease I (NEB). The full length cDNA library was amplified using a single primer and purified with AMPure beads (Beckman Coulter). The sequencing library was prepared by tagmentation of 10 ng full length cDNA with an in house made Tn5 transposase at 55 °C for 9 minutes^74^. Tagmented DNA was purified with DNA Clean and Concentration kit and PCR amplified using NEBNext High-Fidelity 2X PCR Master Mix (NEB) with an i7 adapter identical to Illumina Nextera and custom i5 (Microsynth). The PCR reaction was then purified twice with AMPure beads and the average fragment size of the library was evaluated using Fragment Analyzer (Advanced Analytical) prior to paired-end sequencing with NextSeq 500 (Illumina). This dataset has been deposited in ArrayExpress, currently awaiting the provision of an accession number.

### Barcoded mRNA-seq preprocessing of Illumina reads

Reads from barcoded mRNA-seq experiments have two barcodes, corresponding to the two levels of multiplexing. The first one is common to standard protocols and is used to separate the libraries. The second is specific to the barcoded mRNA-seq protocol and is used to separate the multiplexed samples from the bulk data. The first demultiplexing step was performed with the Illumina BaseSpace platform, while the second was performed using custom scripts. FastQ files were evaluated using FastQC and further mapped to the mm10 genome (GRCm38 r84 from Ensembl) using STAR (with default parameters). Samples that did not aggregate enough reads were excluded from further analysis (<1 M). The count table was then obtained using HTseq (47,729 genes for 23 samples). Genes with a count per million (cpm) greater than 1 for more than 20 samples were retained, providing a filtered dataset of 12,380 expressed genes across 23 samples. Raw counts were then normalized using the *voom* package in R. Hierarchical clustering (Fig. 4) was performed using the *ape* package in R.

### Network construction and PathVisio analysis

We used a recent review^6^ to compile a brown adipogenic gene regulatory network and displayed it using PathVisio^75^. We then superimposed the following two sets of information on the GRN (1) Expression information (log2 fold changes upon *Nfia* knockdown at Day 6) (2) NFI binding site information for each gene within the network in Fig. 6.

## Additional Information

**How to cite this article**: Pradhan, R. N. *et al*. Dissecting the brown adipogenic regulatory network using integrative genomics. *Sci. Rep.* **7**, 42130; doi: 10.1038/srep42130 (2017).

**Publisher's note:** Springer Nature remains neutral with regard to jurisdictional claims in published maps and institutional affiliations.

## Supplementary Material

Supplementary Figures and Tables
