# Supplementary material for: Dissecting the brown adipogenic regulatory network using integrative genomics
Source: Sci Rep. 2017 Feb 9;7:42130. doi: 10.1038/srep42130 (PMC5299609; doi:10.1038/srep42130)
Supplement: Supplementary Figures and Tables [file srep42130-s1.pdf]

Dissecting the brown adipogenic regulatory network using integrative genomics

Rachana N. Pradhan[1,2], Johannes J. Bues[1,2], Vincent Gardeux[1,2], Petra C. Schwalie[1,2], Daniel Alpern[1,2], Wanze Chen[1,2], Julie Russeil[1,2], Sunil K. Raghav[3], Bart Deplancke[1,2,*]

[1]Institute of Bioengineering, École Polytechnique Fédérale de Lausanne (EPFL), CH-1015 Lausanne, Switzerland

[2]Swiss Institute of Bioinformatics, CH-1015 Lausanne, Switzerland

[3]ILS Bhubhaneshwar, Orissa, India

[*]To whom correspondence should be addressed: bart.deplancke@epfl.ch

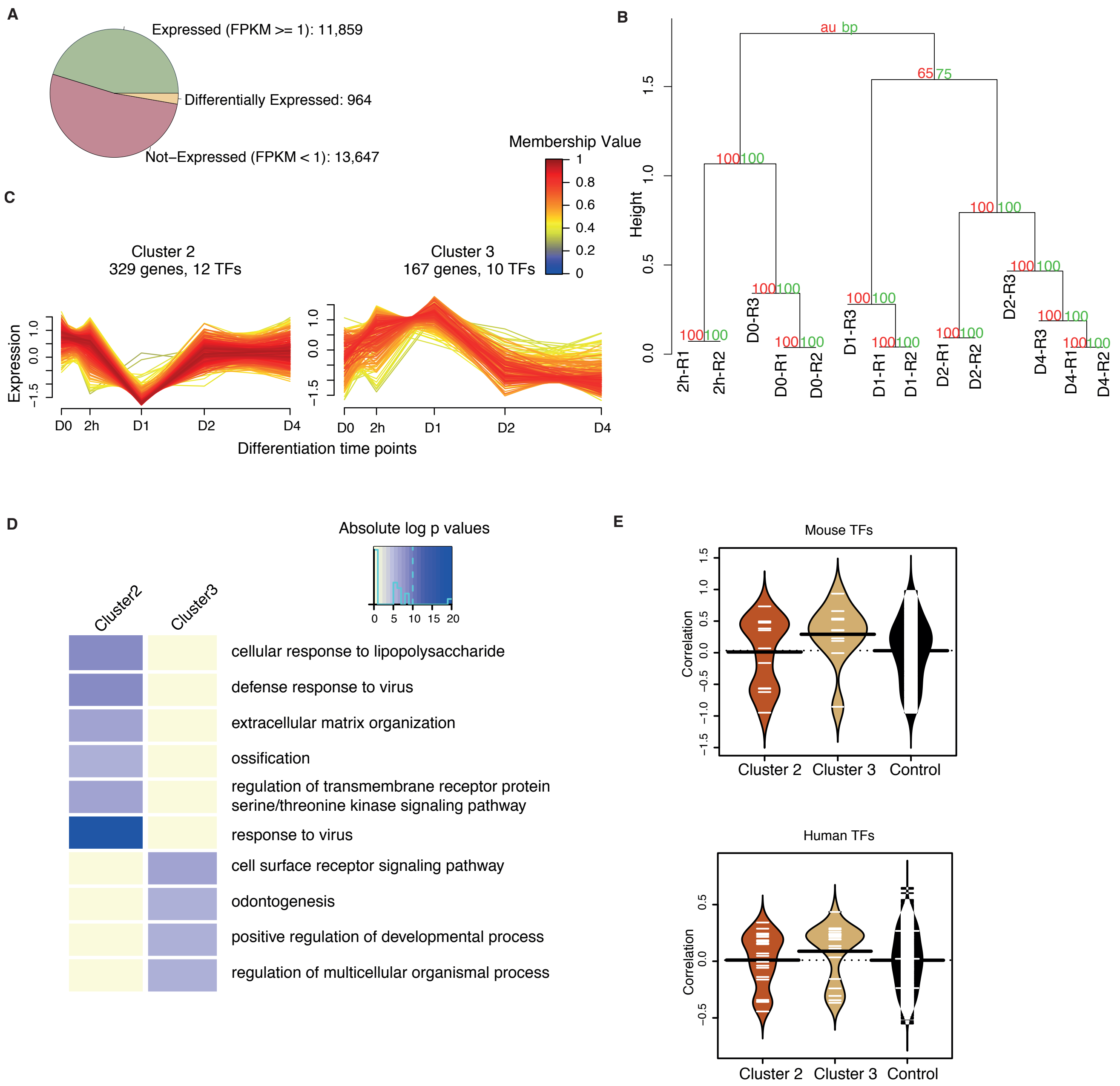
A
Expressed (FPKM >= 1): 11,859
Differentially Expressed: 964
Not−Expressed (FPKM < 1): 13,647
B
au bp
Height
C
Membership Value
Cluster 2
329 genes, 12 TFs
Cluster 3
167 genes, 10 TFs
Expression
Differentiation time points
D0 2h D1 D2 D4
2h-R1 2h-R2 D0-R3 D0-R1 D0-R2 D1-R3 D1-R1 D1-R2 D2-R1 D2-R2 D2-R3 D4-R3 D4-R1 D4-R2
D
Absolute log p values
Cluster2 Cluster3
cellular response to lipopolysaccharide
defense response to virus
extracellular matrix organization
ossification
regulation of transmembrane receptor protein serine/threonine kinase signaling pathway
response to virus
cell surface receptor signaling pathway
odontogenesis
positive regulation of developmental process
regulation of multicellular organismal process
E
Mouse TFs
Human TFs
Correlation
Cluster 2 Cluster 3 Control

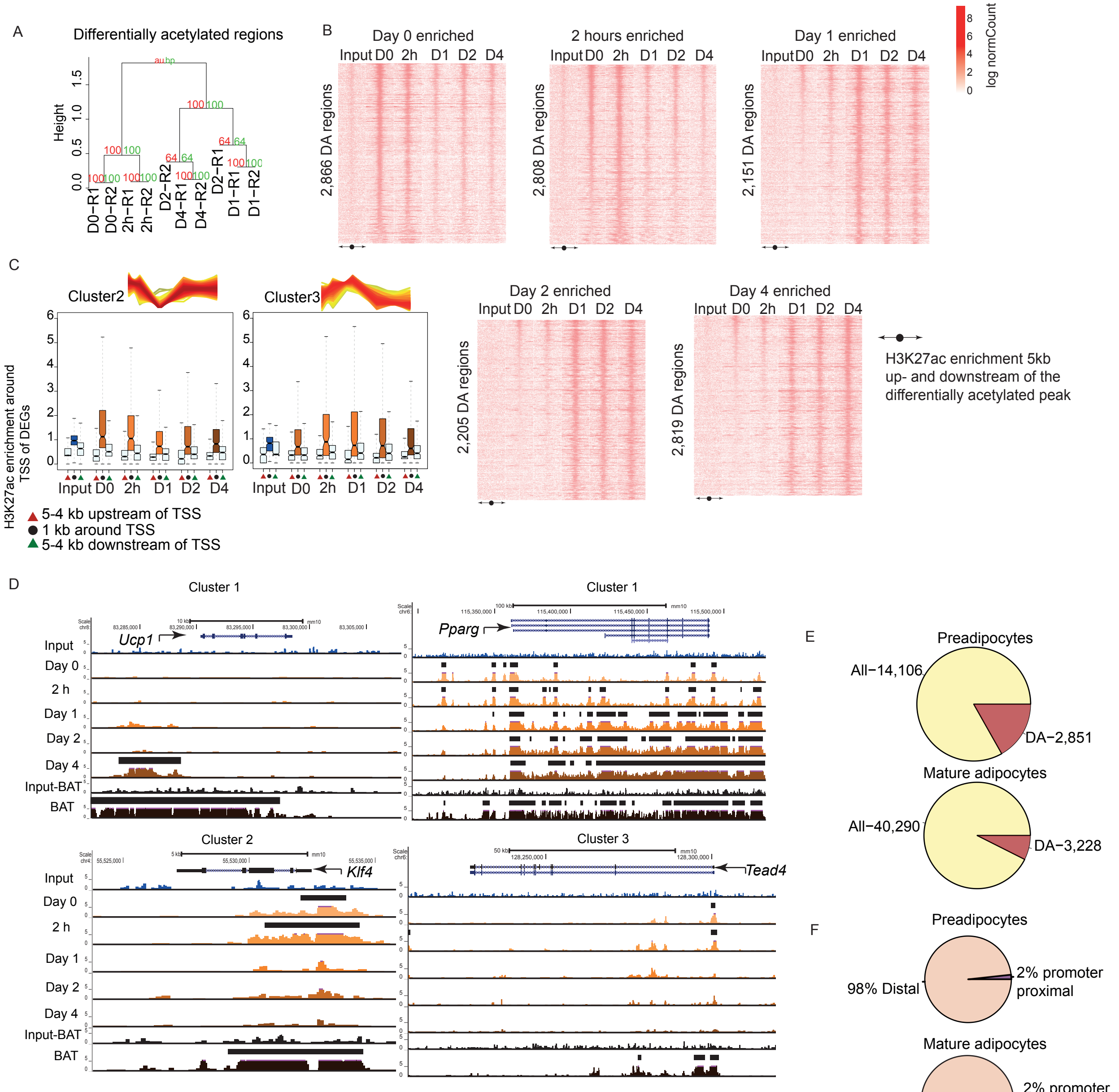

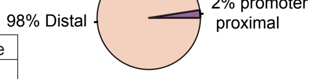

G

Preadipocytes

GO Term	p-value
regulation of developmental growth	0.00071
cell fate commitment	0.00107
regulation of cell proliferation	0.00135
cell morphogenesis involved in differentiation	0.00164
actin filament based process	0.00871

Mature adipocytes

GO Term	p-value
brown fat cell differentiation	$<10^{-11}$
diet induced thermogenesis	0.00026
response to insulin	0.00028
triglyceride biosynthetic process	0.0024
fatty acid homeostasis	0.00379

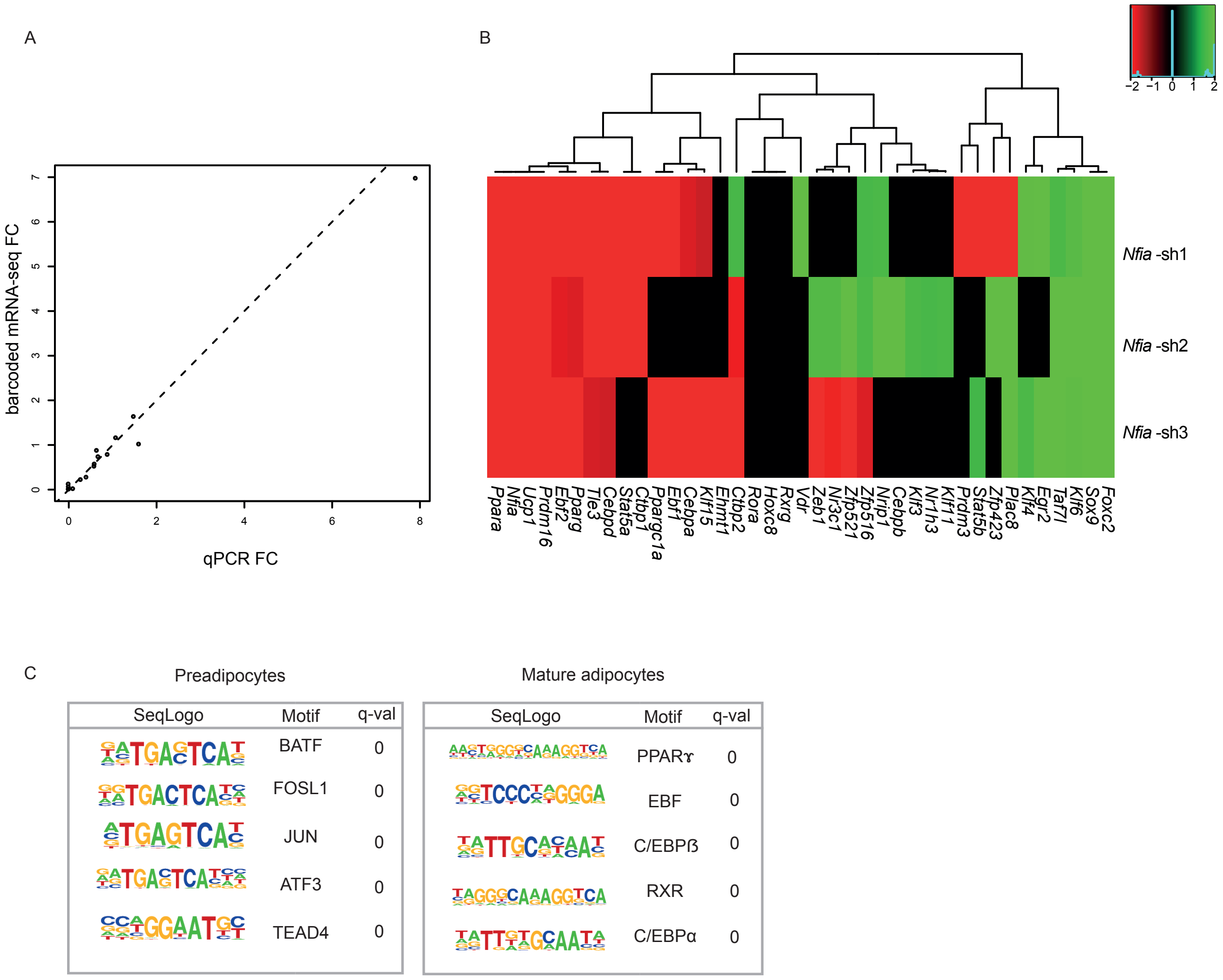
A
B
C
barcoded mRNA-seq FC
qPCR FC
0
1
2
3
4
5
6
7
8
−2 −1 0 1 2
Nfia -sh1
Nfia -sh2
Nfia -sh3
Ppara
Nfia
Ucp1
Prdm16
Ebf2
Pparg
Tle3
Cebpd
Stat5a
Ctbp1
Ppargc1a
Ebf1
Cebpa
Klf15
Ehmt1
Ctbp2
Rora
Hoxc8
Rxrg
Vdr
Zeb1
Nr3c1
Zfp521
Zfp516
Nrip1
Cebpb
Klf3
Nr1h3
Klf11
Prdm3
Stat5b
Zfp423
Plac8
Klf4
Egr2
Taf7l
Klf6
Sox9
Foxc2
Preadipocytes
SeqLogo
Motif
q-val
BATF
0
FOSL1
0
JUN
0
ATF3
0
TEAD4
0
Mature adipocytes
SeqLogo
Motif
q-val
PPARɣ
0
EBF
0
C/EBPß
0
RXR
0
C/EBPα
0

A

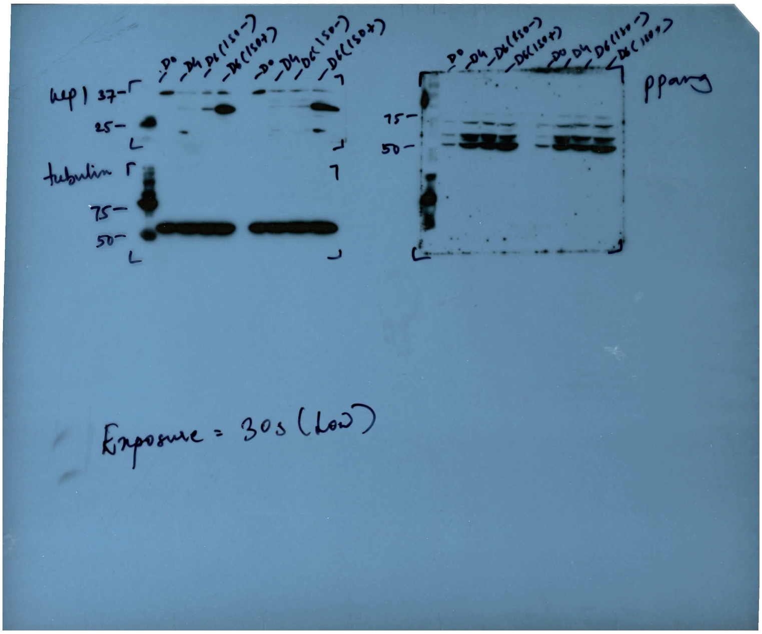
Exposure = 30s (Low)

B

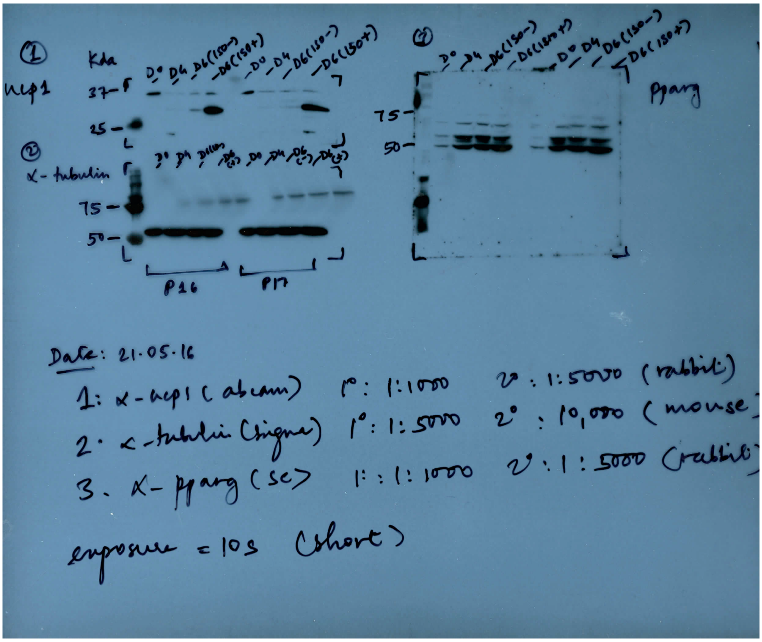
Date: 21.05.16
1: α-ucp1 (abcam) 1°: 1:1000 2°: 1:5000 (rabbit)
2. α-tubulin (sigma) 1°: 1:5000 2°: 10,000 (mouse)
3. α-pparg (sc) 1°: 1:1000 2°: 1:5000 (rabbit)
exposure = 10s (short)

TF-Cluster 1			
**Ensembl_gene_id**	**MEM.SHIP**	**Gene**	**Family**
ENSMUSG00000000440	0.938396902	Pparg	PPAR receptor
ENSMUSG00000000942	0.894532083	Hoxa4	Homeobox
ENSMUSG00000002108	0.916626508	Nr1h3	Ecdystd receptor
ENSMUSG00000003051	0.879524411	Elf3	ETS
ENSMUSG00000004043	0.771996741	Stat5a	STAT
ENSMUSG00000005373	0.908177075	Mlxipl	bHLH
ENSMUSG00000015843	0.909453196	Rxrg	Thyroid hormone receptor
ENSMUSG00000018554	0.928143338	Ybx2	CSD
ENSMUSG00000020538	0.880969352	Srebf1	bHLH
ENSMUSG00000021356	0.593390474	Irf4	IRF
ENSMUSG00000022383	0.895487998	Ppara	PPAR receptor
ENSMUSG00000022508	0.550584671	Bcl6	ZBTB
ENSMUSG00000022987	0.501336804	Zfp641	zf-C2H2
ENSMUSG00000028150	0.662219695	Rorc	Thyroid hormone receptor
ENSMUSG00000028565	0.580531709	Nfia	CTF/NFI
ENSMUSG00000030087	0.813929276	Klf15	zf-C2H2
ENSMUSG00000034957	0.726919853	Cebpa	C/EBP
ENSMUSG00000039087	0.801841035	Rreb1	zf-C2H2
ENSMUSG00000045333	0.920065379	Zfp423	zf-C2H2
ENSMUSG00000046470	0.59210733	Sox18	HMG
ENSMUSG00000059824	0.5899506	Dbp	TF_bZIP
ENSMUSG00000066687	0.498157771	Zbtb16	ZBTB
ENSMUSG00000067929	0.332985651	Gm10226	zf-C2H2
ENSMUSG00000068551	0.766965764	Zfp467	zf-C2H2

TF-Cluster 2			
**Ensembl_gene_id**	**MEM.SHIP**	**Gene**	**Family**
ENSMUSG00000002325	0.822872592	Irf9	IRF
ENSMUSG00000003032	0.4720978	Klf4	zf-C2H2
ENSMUSG00000015709	0.928617603	Arnt2	bHLH
ENSMUSG00000017724	0.553709959	Etv4	ETS
ENSMUSG00000020679	0.390572502	Hnf1b	Homeobox
ENSMUSG00000025498	0.793876317	Irf7	IRF
ENSMUSG00000027230	0.731720781	Creb3l1	TF_bZIP
ENSMUSG00000028358	0.862699381	Zfp618	zf-C2H2
ENSMUSG00000036036	0.759188029	Zfp57	zf-C2H2
ENSMUSG00000040270	0.742078525	Bach2	TF_bZIP
ENSMUSG00000043903	0.602917746	Gm22	zf-C2H2
ENSMUSG00000071064	0.642167681	Zfp827	zf-C2H2
ENSMUSG00000074622	0.28731178	Mafb	TF_bZIP

TF-Cluster 3

Ensembl_gene_id	MEM.SHIP	Gene	Family
ENSMUSG00000001497	0.640669816	Pax9	PAX
ENSMUSG00000001510	0.461887017	Dlx3	Homeobox
ENSMUSG00000022346	0.592230335	Myc	bHLH
ENSMUSG00000024912	0.474488038	Fosl1	TF_bZIP
ENSMUSG00000030353	0.550976621	Tead4	TEA
ENSMUSG00000031431	0.599520078	Tsc22d3	TSC22
ENSMUSG00000036192	0.583887933	Rorb	ROR receptor
ENSMUSG00000042821	0.781413925	Snai1	zf-C2H2
ENSMUSG00000056501	0.441546917	Cebpb	C/EBP
ENSMUSG00000070691	0.706162643	Runx3	Runt

TF-Cluster 4

Ensembl_gene_id	MEM.SHIP	Gene	Family
ENSMUSG00000005148	0.495339769	Klf5	zf-C2H2
ENSMUSG00000007805	0.896954558	Twist2	bHLH
ENSMUSG00000015053	0.662582032	Gata2	zf-GATA
ENSMUSG00000020644	0.621877604	Id2	bHLH
ENSMUSG00000021379	0.459644584	Id4	bHLH
ENSMUSG00000022479	0.921474684	Vdr	Thyroid hormone receptor
ENSMUSG00000022500	0.831513713	Litaf	zf-LITAF-like
ENSMUSG00000022895	0.879759352	Ets2	ETS
ENSMUSG00000022952	0.443317539	Runx1	Runt
ENSMUSG00000023034	0.660611281	Nr4a1	Nuclear orphan receptor
ENSMUSG00000023391	0.649215479	Dlx2	Homeobox
ENSMUSG00000025880	0.714194505	Smad7	MH1
ENSMUSG00000026628	0.45434623	Atf3	TF_bZIP
ENSMUSG00000026630	0.548848944	Batf3	TF_bZIP
ENSMUSG00000026826	0.914638161	Nr4a2	Nuclear orphan receptor
ENSMUSG00000028023	0.385606084	Pitx2	Homeobox
ENSMUSG00000028341	0.918208636	Nr4a3	Nuclear orphan receptor
ENSMUSG00000029135	0.925841733	Fosl2	TF_bZIP
ENSMUSG00000030067	0.719916363	Foxp1	Fork head
ENSMUSG00000031734	0.778356028	Irx3	Homeobox
ENSMUSG00000031737	0.585923635	Irx5	Homeobox
ENSMUSG00000034161	0.448687108	Scx	bHLH
ENSMUSG00000034762	0.781042023	Glis1	zf-C2H2
ENSMUSG00000037868	0.577717425	Egr2	zf-C2H2
ENSMUSG00000038402	0.877802201	Foxf2	Fork head
ENSMUSG00000038518	0.387291987	Jarid2	ARID
ENSMUSG00000038648	0.913481444	Creb3l2	TF_bZIP
ENSMUSG00000041911	0.672869879	Dlx1	Homeobox
ENSMUSG00000042622	0.83916131	Maff	TF_bZIP
ENSMUSG00000042812	0.925026361	Foxf1a	Fork head
ENSMUSG00000046714	0.754994464	Foxc2	Fork head
ENSMUSG00000051341	0.782004205	Zfp52	zf-C2H2
ENSMUSG00000052040	0.415575526	Klf13	zf-C2H2
ENSMUSG00000052837	0.645118303	Junb	TF_bZIP
ENSMUSG00000056592	0.501481298	Zfp658	zf-C2H2
ENSMUSG00000056749	0.506637855	Nfil3	TF_bZIP
ENSMUSG00000056758	0.61663579	Hmga2	HMGI/HMGY
ENSMUSG00000063889	0.80210794	Crem	TF_bZIP
ENSMUSG00000070643	0.747506056	Sox13	HMG
ENSMUSG00000078302	0.753054892	Foxd1	Fork head

Supplementary Table 2

Motif Name (size 1000)-Preadipocytes
JUN_MOUSE.H10MO.A
JUND_MOUSE.H10MO.A
Atf3(bZIP)/GBM-ATF3-ChIP-Seq(GSE33912)/Homer
BATF(bZIP)/Th17-BATF-ChIP-Seq(GSE39756)/Homer
FOSL1_MOUSE.H10MO.C
FOSL2_MOUSE.H10MO.A
AP-1(bZIP)/ThioMac-PU.1-ChIP-Seq(GSE21512)/Homer
Jun-AP1(bZIP)/K562-cJun-ChIP-Seq(GSE31477)/Homer
BATF_MOUSE.H10MO.B
JUNB_MOUSE.H10MO.B
SMRC1_MOUSE.H10MO.B
FOSB_MOUSE.H10MO.C
Bach2(bZIP)/OCILy7-Bach2-ChIP-Seq(GSE44420)/Homer
TEAD4(TEA)/Tropoblast-Tead4-ChIP-Seq(GSE37350)/Homer
TEAD(TEA)/Fibroblast-PU.1-ChIP-Seq(Unpublished)/Homer
NFE2_MOUSE.H10MO.A
MAFK_MOUSE.H10MO.S
BACH1_MOUSE.H10MO.C
NF2L2_MOUSE.H10MO.D
NF-E2(bZIP)/K562-NFE2-ChIP-Seq(GSE31477)/Homer
Bach1(bZIP)/K562-Bach1-ChIP-Seq(GSE31477)/Homer
Nrf2(bZIP)/Lymphoblast-Nrf2-ChIP-Seq(GSE37589)/Homer
Mef2c(MADS)/GM12878-Mef2c-ChIP-Seq(GSE32465)/Homer
TEAD1_MOUSE.H10MO.D
MEF2D_MOUSE.H10MO.C
MafK(bZIP)/C2C12-MafK-ChIP-Seq(GSE36030)/Homer
Ets1-distal(ETS)/CD4+-PolII-ChIP-Seq(Barski et al.)/Homer
RUNX1(Runt)/Jurkat-RUNX1-ChIP-Seq(GSE29180)/Homer
COE1_MOUSE.H10MO.C
FEV_MOUSE.H10MO.C
RUNX1_MOUSE.H10MO.B
MAFG_MOUSE.H10MO.D
MEF2A_MOUSE.H10MO.B
Stat3(Stat)/mES-Stat3-ChIP-Seq(GSE11431)/Homer
ERG(ETS)/VCaP-ERG-ChIP-Seq(GSE14097)/Homer
RUNX(Runt)/HPC7-Runx1-ChIP-Seq(GSE22178)/Homer
EBF(EBF)/proBcell-EBF-ChIP-Seq(GSE21978)/Homer
NF1:FOXA1/LNCAP-FOXA1-ChIP-Seq(GSE27824)/Homer
EBF1(EBF)/Near-E2A-ChIP-Seq(GSE21512)/Homer
NFIA_MOUSE.H10MO.C
RUNX-AML(Runt)/CD4+-PolII-ChIP-Seq(Barski et al.)/Homer
NF1-halfsite(CTF)/LNCaP-NF1-ChIP-Seq(Unpublished)/Homer
ELK1_MOUSE.H10MO.B

EWS:ERG-fusion(ETS)/CADO_ES1-EWS:ERG-ChIP-Seq(SRA014231)/Home
EWS:FLI1-fusion(ETS)/SK_N_MC-EWS:FLI1-ChIP-Seq(SRA014231)/Homer
SPDEF(ETS)/VCaP-SPDEF-ChIP-Seq(SRA014231)/Homer
Mef2a(MADS)/HL1-Mef2a.biotin-ChIP-Seq(GSE21529/Homer
PEBB_MOUSE.H10MO.C
ETS1(ETS)/Jurkat-ETS1-ChIP-Seq(GSE17954)/Homer
Fli1(ETS)/CD8-FLI-ChIP-Seq(GSE20898)/Homer
ETS1_MOUSE.H10MO.B
ETV1(ETS)/GIST48-ETV1-ChIP-Seq(GSE22441)/Homer
RUNX2_MOUSE.H10MO.B
RUNX2(Runt)/PCa-RUNX2-ChIP-Seq(GSE33889)/Homer
ELF1_MOUSE.H10MO.B
STA5A_MOUSE.H10MO.A
MEF2C_MOUSE.H10MO.B
Tlx?(NR)/NPC-H3K4me1-ChIP-Seq(GSE16256)/Homer
GABPA(ETS)/Jurkat-GABPa-ChIP-Seq(GSE17954)/Homer
ETV4_MOUSE.H10MO.B
NFIA_MOUSE.H10MO.S
FLI1_MOUSE.H10MO.A
GABP1_MOUSE.H10MO.C
TEAD4_MOUSE.H10MO.D
STA5B_MOUSE.H10MO.C
Atf4(bZIP)/MEF-Atf4-ChIP-Seq(GSE35681)/Homer
MAFK_MOUSE.H10MO.A
NF1(CTF)/LNCAP-NF1-ChIP-Seq(Unpublished)/Homer
MITF_MOUSE.H10MO.C
ERG_MOUSE.H10MO.A
USF2_MOUSE.H10MO.A
NFAC1_MOUSE.H10MO.A
STAT4(Stat)/CD4-Stat4-ChIP-Seq(GSE22104)/Homer
Chop(bZIP)/MEF-Chop-ChIP-Seq(GSE35681)/Homer
STAT3_MOUSE.H10MO.B
NFAC1_MOUSE.H10MO.S
EHF(ETS)/LoVo-EHF-ChIP-Seq(GSE49402)/Homer
NFAT:AP1/Jurkat-NFATC1-ChIP-Seq(Jolma et al.)/Homer
NFkB-p65-Rel(RHD)/LPS-exp(GSE23622)/Homer
NF2L1_MOUSE.H10MO.C
STAT5(Stat)/mCD4+-Stat5-ChIP-Seq(GSE12346)/Homer
Usf2(HLH)/C2C12-Usf2-ChIP-Seq(GSE36030)/Homer
Fox:Ebox(Forkhead:HLH)/Panc1-Foxa2-ChIP-Seq(GSE47459)/Homer
CEBPD_MOUSE.H10MO.B
CEBPB_MOUSE.H10MO.A
EHF_MOUSE.H10MO.A
HOXA9(Homeobox)/HSC-Hoxa9-ChIP-Seq(GSE33509)/Homer
Bcl6(Zf)/Liver-Bcl6-ChIP-Seq(GSE31578)/Homer
MafA(bZIP)/Islet-MafA-ChIP-Seq(GSE30298)/Homer

STAT1(Stat)/HelaS3-STAT1-ChIP-Seq(GSE12782)/Homer
USF1(HLH)/GM12878-Usf1-ChIP-Seq(GSE32465)/Homer
CEBPA_MOUSE.H10MO.B
Smad3(MAD)/NPC-Smad3-ChIP-Seq(GSE36673)/Homer
STAT4_MOUSE.H10MO.A
Smad4(MAD)/ESC-SMAD4-ChIP-Seq(GSE29422)/Homer
USF1_MOUSE.H10MO.A
IRF2_MOUSE.H10MO.C
Elk4(ETS)/Hela-Elk4-ChIP-Seq(GSE31477)/Homer
NFAC4_MOUSE.H10MO.C
CEBP:AP1(bZIP)/ThioMac-CEBPb-ChIP-Seq(GSE21512)/Homer
STAT6/Macrophage-Stat6-ChIP-Seq(GSE38377)/Homer
GABPA_MOUSE.H10MO.A
AP-2gamma(AP2)/MCF7-TFAP2C-ChIP-Seq(GSE21234)/Homer
Stat3+il21(Stat)/CD4-Stat3-ChIP-Seq(GSE19198)/Homer
Pdx1(Homeobox)/Islet-Pdx1-ChIP-Seq(SRA008281)/Homer
Ap4(HLH)/AML-Tfap4-ChIP-Seq(GSE45738)/Homer
ELF1(ETS)/Jurkat-ELF1-ChIP-Seq(SRA014231)/Homer
Elk1(ETS)/Hela-Elk1-ChIP-Seq(GSE31477)/Homer
FOXP2_MOUSE.H10MO.A
Hoxc9(Homeobox)/Ainv15-Hoxc9-ChIP-Seq(GSE21812)/Homer
IRF7_MOUSE.H10MO.C
ESR2_MOUSE.H10MO.S
STAT6(Stat)/CD4-Stat6-ChIP-Seq(GSE22104)/Homer
DBP_MOUSE.H10MO.B
KLF8_MOUSE.H10MO.C
SP1_MOUSE.H10MO.S
STAT6_MOUSE.H10MO.C
TF65_MOUSE.H10MO.S
ATF3_MOUSE.H10MO.A
NFkB-p65(RHD)/GM12787-p65-ChIP-Seq(GSE19485)/Homer
PHA-4(Forkhead)/cElegans-Embryos-PHA4-ChIP-Seq(modEncode)/Homer
FOXA1(Forkhead)/LNCAP-FOXA1-ChIP-Seq(GSE27824)/Homer
BCL6_MOUSE.H10MO.C
HRE(HSF)/Striatum-HSF1-ChIP-Seq(GSE38000)/Homer
ELK4_MOUSE.H10MO.B
ETS(ETS)/Promoter/Homer
E2F6_MOUSE.H10MO.A
FOS_MOUSE.H10MO.A
RELB_MOUSE.H10MO.C
ELF5(ETS)/T47D-ELF5-ChIP-Seq(GSE30407)/Homer

ZN143_MOUSE.H10MO.D
SCL(HLH)/HPC7-Scl-ChIP-Seq(GSE13511)/Homer
MYF6_MOUSE.H10MO.C
ATF2_MOUSE.H10MO.B

MyoD(HLH)/Myotube-MyoD-ChIP-Seq(GSE21614)/Homer
FOXA1(Forkhead)/MCF7-FOXA1-ChIP-Seq(GSE26831)/Homer
NFAT5_MOUSE.H10MO.D
AARE(HLH)/mES-cMyc-ChIP-Seq/Homer
NFAC3_MOUSE.H10MO.B
BMYB(HTH)/Hela-BMYB-ChIPSeq(GSE27030)/Homer
EVX1_MOUSE.H10MO.C
Cbf1(bHLH)/Yeast-Cbf1-ChIP-Seq(GSE29506)/Homer
p53(p53)/mES-cMyc-ChIP-Seq(GSE11431)/Homer
FOXA1_MOUSE.H10MO.B
E-box/Drosophila-Promoters/Homer
ELF2_MOUSE.H10MO.C
Rfx1(HTH)/NPC-H3K4me1-ChIP-Seq(GSE16256)/Homer
GFI1_MOUSE.H10MO.C

TF65_MOUSE.H10MO.C
NR1D1_MOUSE.H10MO.D
Foxa2(Forkhead)/Liver-Foxa2-ChIP-Seq(GSE25694)/Homer
REL_MOUSE.H10MO.C
Atoh1(bHLH)/Cerebellum-Atoh1-ChIP-Seq(GSE22111)/Homer
STAT2_MOUSE.H10MO.B
Rbpj1(?)/Panc1-Rbpj1-ChIP-Seq(GSE47459)/Homer
THB_MOUSE.H10MO.S
MAX_MOUSE.H10MO.A
CDX4_MOUSE.H10MO.A
Gfi1b(Zf)/HPC7-Gfi1b-ChIP-Seq(GSE22178)/Homer
OLIG2_MOUSE.H10MO.B
Reverb(NR/DR2)/BLRP(RAW)-Reverba-ChIP-Seq(GSE45914)/Homer
KLF4_MOUSE.H10MO.C
PU.1(ETS)/ThioMac-PU.1-ChIP-Seq(GSE21512)/Homer
ZBT18_MOUSE.H10MO.D
c-Jun-CRE(bZIP)/K562-cJun-ChIP-Seq(GSE31477)/Homer
Arnt:Ahr(bHLH)/MCF7-Arnt-ChIP-Seq(Lo et al.)/Homer
PAX2_MOUSE.H10MO.D
Nur77(NR)/K562-NR4A1-ChIP-Seq(GSE31363)/Homer
FOXP1(Forkhead)/H9-FOXP1-ChIP-Seq(GSE31006)/Homer
PIF5ox(bHLH)/Arabidopsis-PIF5ox-ChIP-Seq(GSE35062)/Homer
TFE3_MOUSE.H10MO.C
IRF3_MOUSE.H10MO.C
Foxh1(Forkhead)/hESC-FOXH1-ChIP-Seq(GSE29422)/Homer
FOXC1_MOUSE.H10MO.C
Tcf12(HLH)/GM12878-Tcf12-ChIP-Seq(GSE32465)/Homer
Hoxb4(Homeobox)/ES-Hoxb4-ChIP-Seq(GSE34014)/Homer
FOXD1_MOUSE.H10MO.D
E2F4_MOUSE.H10MO.C
AP2C_MOUSE.H10MO.A

X-box(HTH)/NPC-H3K4me1-ChIP-Seq(GSE16256)/Homer
BMAL1_MOUSE.H10MO.C
FOXJ2_MOUSE.H10MO.C
RFX2_MOUSE.H10MO.C
FOXA2_MOUSE.H10MO.B
HLTF_MOUSE.H10MO.D
AP2A_MOUSE.H10MO.A
RFX(HTH)/K562-RFX3-ChIP-Seq(SRA012198)/Homer
ELF3_MOUSE.H10MO.D
CEBPG_MOUSE.H10MO.C
PAX5_MOUSE.H10MO.S
EGR4_MOUSE.H10MO.D
HNF4G_MOUSE.H10MO.C
Smad2(MAD)/ES-SMAD2-ChIP-Seq(GSE29422)/Homer
GFI1B_MOUSE.H10MO.C
NFAT(RHD)/Jurkat-NFATC1-ChIP-Seq(Jolma et al.)/Homer
LIN-39(Homeobox)/cElegans.L3-LIN39-ChIP-Seq(modEncode)/Homer
Maz(Zf)/HepG2-Maz-ChIP-Seq(GSE31477)/Homer
IRF8_MOUSE.H10MO.D
HSF1_MOUSE.H10MO.A
ELF5_MOUSE.H10MO.D
MAFB_MOUSE.H10MO.D
XBP1_MOUSE.H10MO.C
ZN148_MOUSE.H10MO.D
Unknown4/Drosophila-Promoters/Homer
MEIS1_MOUSE.H10MO.C
Pitx1(Homeobox)/Chicken-Pitx1-ChIP-Seq(GSE38910)/Homer

RFX1_MOUSE.H10MO.C
HAND1_MOUSE.H10MO.D
Unknown-ESC-element/mES-Nanog-ChIP-Seq(GSE11724)/Homer
Rfx2(HTH)/LoVo-RFX2-ChIP-Seq(GSE49402)/Homer
PAX5-shortForm(Paired/Homeobox)/GM12878-PAX5-ChIP-Seq(GSE32465)/[illegible]
RXRB_MOUSE.H10MO.C
ETV5_MOUSE.H10MO.D
NFIL3_MOUSE.H10MO.C
SP1_MOUSE.H10MO.A

Consensus	P-value	Log P-value	q-value (Benjamini)
MTGAGTCAY	1e-313	-7.23E+02	0
GATGAGTCATC	1.00E-300	-6.93E+02	0
DATGASTCATHN	1.00E-287	-6.62E+02	0
DATGASTCAT	1.00E-279	-6.43E+02	0
DRTGACTCAYN	1.00E-278	-6.41E+02	0
GGATGAGTCAY	1.00E-276	-6.38E+02	0
VTGACTCATC	1.00E-267	-6.16E+02	0
GATGASTCATCN	1.00E-266	-6.13E+02	0
MTGAGTCATW	1.00E-255	-5.89E+02	0
DVTGAGTCAK	1.00E-240	-5.54E+02	0
VTGAGTCAB	1.00E-212	-4.89E+02	0
GATGAGTCAG	1.00E-144	-3.33E+02	0
TGCTGAGTCA	1.00E-107	-2.47E+02	0
CCWGGAATGY	1.00E-98	-2.27E+02	0
YCWGGAATGY	1.00E-91	-2.10E+02	0
RSVATGACTCAGCA	1.00E-62	-1.43E+02	0
TGCTGAGTCA	1.00E-59	-1.37E+02	0
TGCTGAGTCAYGGT	1.00E-49	-1.13E+02	0
VRTGACTCAGCA	1.00E-48	-1.12E+02	0
GATGACTCAGCA	1.00E-40	-9.26E+01	0
AWWNTGCTGAGTCAT	1.00E-38	-8.89E+01	0
HTGCTGAGTCAT	1.00E-35	-8.10E+01	0
DCYAAAAATAGM	1.00E-25	-5.98E+01	0
CACATTCCWGMKCH	1.00E-25	-5.80E+01	0
GCTATTTTTAGV	1.00E-21	-4.87E+01	0
GCTGASTCAGCA	1.00E-20	-4.83E+01	0
MACAGGAAGT	1.00E-20	-4.75E+01	0
AAACCACARM	1.00E-19	-4.57E+01	0
GTCCCTGGGGASY	1.00E-19	-4.41E+01	0
CAGGAARTDA	1.00E-18	-4.36E+01	0
TYTGTGGTTA	1.00E-18	-4.22E+01	0
AGTCATK	1.00E-17	-4.05E+01	0
RDKCTATTTWTAGM	1.00E-17	-3.98E+01	0
CTTCCGGGAA	1.00E-17	-3.98E+01	0
ACAGGAAGTG	1.00E-16	-3.89E+01	0
SAAACCACAG	1.00E-16	-3.89E+01	0
DGTCCCYRGGGA	1.00E-16	-3.88E+01	0
WNTGTTTRYTTTGGCA	1.00E-16	-3.86E+01	0
GTCCCCWGGGGA	1.00E-15	-3.64E+01	0
TTGGCANGNTGCCAR	1.00E-15	-3.62E+01	0
GCTGTGGTTW	1.00E-15	-3.51E+01	0
YTGCCAAG	1.00E-14	-3.34E+01	0
RCCGGAAGT	1.00E-13	-3.20E+01	0

ATTTCCTGTN	1.00E-13	-3.16E+01	0
VACAGGAAAT	1.00E-13	-3.13E+01	0
ASWTCCTGBT	1.00E-13	-3.10E+01	0
CYAAAAATAG	1.00E-13	-3.02E+01	0
TYTGTGGTYWB	1.00E-12	-2.94E+01	0
ACAGGAAGTG	1.00E-12	-2.83E+01	0
NRYTTCCGGH	1.00E-12	-2.80E+01	0
ACAGGAAGT	1.00E-12	-2.80E+01	0
AACCGGAAGT	1.00E-12	-2.80E+01	0
YTGTGGTTDGK	1.00E-12	-2.77E+01	0
NWAACCACADNN	1.00E-12	-2.76E+01	0
VCMGGAAGTG	1.00E-11	-2.75E+01	0
WTTTCTTGGAAAT	1.00E-11	-2.72E+01	0
TKCTAWAAATAGM	1.00E-11	-2.68E+01	0
CTGGCAGSCTGCCA	1.00E-11	-2.66E+01	0
RACCGGAAGT	1.00E-11	-2.61E+01	0
SAGGAAGT	1.00E-11	-2.58E+01	0
MTTGGCA	1.00E-11	-2.55E+01	0
SVRACCGGAAGTGGS	1.00E-10	-2.53E+01	0
CCGGAAGTGV	1.00E-10	-2.51E+01	0
AAAAATAKCYCT	1.00E-10	-2.49E+01	0
RTTTCTKRGAA	1.00E-10	-2.46E+01	0
MTGATGCAAT	1.00E-10	-2.46E+01	0
AAAWNTGCTGASTCAGCA	1.00E-10	-2.43E+01	0
CYTGGCABNSTGCCAR	1.00E-10	-2.41E+01	0
RTCATGTGMB	1.00E-10	-2.41E+01	0
ACCGGAARTCC	1.00E-10	-2.39E+01	0
GGTCACGTGRCSS	1.00E-10	-2.38E+01	0
CAATGGAAAWTT	1.00E-10	-2.37E+01	0
NYTTCCWGGAAR	1.00E-10	-2.31E+01	0
ATTGCATCAT	1.00E-09	-2.29E+01	0
YCTCATTTCCCGKAA	1.00E-09	-2.28E+01	0
TGGAAAWTTCCAT	1.00E-09	-2.28E+01	0
AVCAGGAAGT	1.00E-09	-2.24E+01	0
SARTGGAAAAWRTGAGTCAB	1.00E-09	-2.21E+01	0
GGAAATTCCC	1.00E-09	-2.18E+01	0
NATGACT	1.00E-09	-2.13E+01	0
RTTTCTNAGAAA	1.00E-09	-2.09E+01	0
GTCACGTGGT	1.00E-09	-2.09E+01	0
NNNVCTGWGYAAACASN	1.00E-08	-2.03E+01	0
RRTTRBGCAAT	1.00E-08	-2.00E+01	0
KATTGCAYAAT	1.00E-08	-1.98E+01	0
MAACCCGGAAGTA	1.00E-08	-1.93E+01	0
GGCCATAAATCA	1.00E-08	-1.93E+01	0
NNNCTTTCCAGGAAA	1.00E-08	-1.91E+01	0
TGCTGACTCA	1.00E-07	-1.83E+01	0

NATTTCCNGGAAAT	1.00E-07	-1.81E+01	0
SGTCACGTGR	1.00E-07	-1.78E+01	0
TATTKTGCAATA	1.00E-07	-1.78E+01	0
TWGTCTGV	1.00E-07	-1.75E+01	0
TTTCCYGGAAR	1.00E-07	-1.72E+01	0
VBSYGTCTGG	1.00E-07	-1.71E+01	0
GGTCACGTGR	1.00E-07	-1.69E+01	0
SGAAAGTGAAAGYR	1.00E-07	-1.65E+01	0
NRYTTCCGGY	1.00E-07	-1.65E+01	0
AGGAAAAYTW	1.00E-06	-1.57E+01	0
DRTGTTGCAA	1.00E-06	-1.54E+01	0
TTCCKNAGAA	1.00E-06	-1.51E+01	0
GVRCCGGAAGTGV	1.00E-06	-1.51E+01	0
SCCTSAGGSCAW	1.00E-06	-1.51E+01	0
SVYTTCCNGGAARB	1.00E-06	-1.50E+01	0
YCATYAATCA	1.00E-06	-1.49E+01	0
NAHCAGCTGD	1.00E-06	-1.47E+01	0
AVCCGGAAGT	1.00E-06	-1.47E+01	0
HACTTCCGGY	1.00E-06	-1.44E+01	0
BTGTTTACW	1.00E-06	-1.40E+01	0
GGCCATAAATCA	1.00E-05	-1.32E+01	0
GAAASYGAAA	1.00E-05	-1.31E+01	0
AGGTCAS	1.00E-05	-1.30E+01	0
ABTTCYYRRGAA	1.00E-05	-1.28E+01	0
RTTATGYAABM	1.00E-05	-1.27E+01	0
CAGGGGGTG	1.00E-05	-1.23E+01	0
GGGGGCGGGGC	1.00E-05	-1.23E+01	0
ABTTCCTGAGAA	1.00E-05	-1.19E+01	0
GGGAATTTCCC	1.00E-05	-1.18E+01	0
GTGACGYMA	1.00E-05	-1.16E+01	0.0001
WGGGGATTTCCC	1.00E-05	-1.16E+01	0.0001
KTGTTTGC	1.00E-05	-1.15E+01	0.0001
WAAGTAAACA	1.00E-04	-1.15E+01	0.0001
WTTCCTRGAAAGC	1.00E-04	-1.15E+01	0.0001
TTCTAGAABNTTCTA	1.00E-04	-1.13E+01	0.0001
VACCGGAAGTV	1.00E-04	-1.13E+01	0.0001
AACCGGAAGT	1.00E-04	-1.12E+01	0.0001
NGGGCGGGAAGG	1.00E-04	-1.08E+01	0.0001
YTCTGATTGGYY	1.00E-04	-1.08E+01	0.0001
GGGGGMTTTCCM	1.00E-04	-1.06E+01	0.0001
ACVAGGAAGT	1.00E-04	-1.06E+01	0.0001
GCRWKGCATKMTGGGAADTVY H	1.00E-04	-1.06E+01	0.0001
AVCAGCTG	1.00E-04	-1.04E+01	0.0002
GCAGSTG	1.00E-04	-1.03E+01	0.0002
VTGACGTCA	1.00E-04	-1.02E+01	0.0002

RRCAGCTGYTSY	1.00E-04	-1.02E+01	0.0002
WAAGTAAACA	1.00E-04	-1.02E+01	0.0002
GTGGAAAANTCCMTK	1.00E-04	-1.01E+01	0.0002
GATTGCATCA	1.00E-04	-1.01E+01	0.0002
TGGAAAAMT	1.00E-04	-1.01E+01	0.0002
NHAACBGYYV	1.00E-04	-1.00E+01	0.0002
TGATTTATGGCYT	1.00E-04	-9.92E+00	0.0003
TCACGTGAYH	1.00E-04	-9.73E+00	0.0003
ACATGCCCGGGCAT	1.00E-04	-9.61E+00	0.0003
TGTTTRCTYWG	1.00E-04	-9.38E+00	0.0004
AACAGCTGTTHN	1.00E-04	-9.31E+00	0.0005
TRNCAGGAAGTRRVT	1.00E-04	-9.27E+00	0.0005
KGTTGCCATGGCAA	1.00E-04	-9.25E+00	0.0005
GCWGTGATTT	1.00E-03	-9.05E+00	0.0006
GGAAWTTCCMNDATTDCNHAAY	1.00E-03	-9.02E+00	0.0006
ARGDVMVWAGGTCAGDGGG	1.00E-03	-8.97E+00	0.0006
CYTGTTTACWYW	1.00E-03	-8.97E+00	0.0006
DGGGGNTTTCCM	1.00E-03	-8.77E+00	0.0007
VNRVCAGCTGGY	1.00E-03	-8.71E+00	0.0008
TCAGTTTCNKTTTCC	1.00E-03	-8.69E+00	0.0008
HTTTCCCASG	1.00E-03	-8.67E+00	0.0008
SGTCAGGTCA	1.00E-03	-8.60E+00	0.0009
GASCACGTGGC	1.00E-03	-8.58E+00	0.0009
TGATTTATGGCY	1.00E-03	-8.56E+00	0.0009
MAATCACTGC	1.00E-03	-8.53E+00	0.0009
RCCAGCTGBTNNCHGSCTGS	1.00E-03	-8.42E+00	0.001
GTRGGTCASTGGGTCA	1.00E-03	-8.40E+00	0.001
WGGGYGKGGCCS	1.00E-03	-8.39E+00	0.001
AGAGGAAGTG	1.00E-03	-8.36E+00	0.001
TCCAGATGTTYSSS	1.00E-03	-8.26E+00	0.0012
ATGACGTCATCY	1.00E-03	-8.25E+00	0.0012
TBGCACGCAA	1.00E-03	-8.19E+00	0.0012
GHTCAGTCATGCGTGACA	1.00E-03	-8.16E+00	0.0013
TGACCTTTNCNT	1.00E-03	-8.14E+00	0.0013
NYYTGTTTACHN	1.00E-03	-8.13E+00	0.0013
BCACGTGVDN	1.00E-03	-8.09E+00	0.0013
RGTCAYGTGR	1.00E-03	-8.07E+00	0.0013
GGAAAGYGAAACBRRAA	1.00E-03	-8.05E+00	0.0014
NNTGTGGATTSS	1.00E-03	-8.01E+00	0.0014
CDTTGTTTACTTAAG	1.00E-03	-8.01E+00	0.0014
VCAGCTGYTG	1.00E-03	-7.99E+00	0.0014
TGATTRATGGCY	1.00E-03	-7.78E+00	0.0017
CWWTGTTTACHTAAG	1.00E-03	-7.74E+00	0.0018
GDGGGCGGGAAR	1.00E-03	-7.74E+00	0.0018
GCCTGRGGC	1.00E-03	-7.71E+00	0.0018

GGTTGCCATGGCAA	1.00E-03	-7.68E+00	0.0019
KGRACACGTGACYC	1.00E-03	-7.44E+00	0.0024
TGTTTRTTTW	1.00E-03	-7.40E+00	0.0024
DGTTGCYAGGSAA	1.00E-03	-7.40E+00	0.0024
TTGTTTACWTWG	1.00E-03	-7.38E+00	0.0025
KANGGCTGSAAM	1.00E-03	-7.30E+00	0.0027
GCCTGRGGC	1.00E-03	-7.22E+00	0.0029
CGGTTGCCATGGCAAC	1.00E-03	-7.18E+00	0.003
GGSAAACAGGAARY	1.00E-03	-7.18E+00	0.003
ATTKTGCAATCTB	1.00E-03	-7.13E+00	0.0031
GGCTGAGG	1.00E-03	-7.07E+00	0.0033
GGSGGCRGGGC	1.00E-03	-7.05E+00	0.0033
GGCCAAAGTCCA	1.00E-03	-7.01E+00	0.0034
CTGTCTGG	1.00E-03	-7.00E+00	0.0034
GCTGWGATTTV	1.00E-03	-6.95E+00	0.0036
ATTTTCCATT	1.00E-03	-6.94E+00	0.0036
ATGATTRATG	1.00E-03	-6.91E+00	0.0037
GGGGGGGG	1.00E-02	-6.90E+00	0.0037
GRGRAAVTGAAASYR	1.00E-02	-6.62E+00	0.0049
RGAANVTTCYAGAA	1.00E-02	-6.51E+00	0.0055
AHAAGGAARTA	1.00E-02	-6.50E+00	0.0055
TGCTGACDS	1.00E-02	-6.48E+00	0.0056
GACGTGKCMTTW	1.00E-02	-6.46E+00	0.0056
GGGGKGGGGGAGGGG	1.00E-02	-6.46E+00	0.0056
AAAAATACCRMA	1.00E-02	-6.44E+00	0.0057
TGACAGYTTTAHG	1.00E-02	-6.28E+00	0.0067
TAATCCCN	1.00E-02	-6.21E+00	0.0071
SCSSCSGTTGCCAKGGCRACG<br>G	1.00E-02	-6.09E+00	0.008
GBGTCTGGMWTT	1.00E-02	-6.02E+00	0.0085
CACAGCAGGGGG	1.00E-02	-6.00E+00	0.0086
GTTGCCATGGCAACM	1.00E-02	-5.99E+00	0.0087
GTCACGCTCSCTGM	1.00E-02	-5.93E+00	0.0092
TSAGGTCACR	1.00E-02	-5.88E+00	0.0096
GHCAGGAAGWAAY	1.00E-02	-5.87E+00	0.0097
GTTAYGTAAYKYMK	1.00E-02	-5.83E+00	0.01
GGGGRGGGGGCGGGGCCRG	1.00E-02	-5.80E+00	0.0103

# of Target Sequences with Motif(of 2851)	% of Target Sequences with Motif
1521	53.35%
1495	52.44%
1667	58.47%
1664	58.37%
1515	53.14%
1439	50.47%
1732	60.75%
925	32.44%
1505	52.79%
1526	53.53%
1465	51.39%
1284	45.04%
572	20.06%
1550	54.37%
1368	47.98%
1121	39.32%
960	33.67%
943	33.08%
1040	36.48%
200	7.02%
186	6.52%
168	5.89%
826	28.97%
1001	35.11%
964	33.81%
466	16.35%
583	20.45%
1506	52.82%
1005	35.25%
1064	37.32%
933	32.73%
541	18.98%
1059	37.14%
773	27.11%
1856	65.10%
1121	39.32%
352	12.35%
149	5.23%
1208	42.37%
822	28.83%
1111	38.97%
1835	64.36%
410	14.38%

1117	39.18%
841	29.50%
1368	47.98%
704	24.69%
1002	35.15%
1344	47.14%
1299	45.56%
1025	35.95%
1617	56.72%
1109	38.90%
1275	44.72%
878	30.80%
1203	42.20%
812	28.48%
623	21.85%
1134	39.78%
1161	40.72%
617	21.64%
731	25.64%
585	20.52%
1128	39.57%
1130	39.64%
428	15.01%
928	32.55%
524	18.38%
829	29.08%
477	16.73%
482	16.91%
1050	36.83%
1325	46.47%
347	12.17%
1077	37.78%
761	26.69%
1621	56.86%
331	11.61%
116	4.07%
558	19.57%
611	21.43%
449	15.75%
1450	50.86%
528	18.52%
464	16.27%
1066	37.39%
1011	35.46%
1764	61.87%
1080	37.88%

526	18.45%
567	19.89%
775	27.18%
2487	87.23%
1234	43.28%
1774	62.22%
501	17.57%
1215	42.62%
586	20.55%
1248	43.77%
1000	35.08%
831	29.15%
607	21.29%
967	33.92%
987	34.62%
1322	46.37%
1358	47.63%
565	19.82%
585	20.52%
942	33.04%
783	27.46%
1267	44.44%
459	16.10%
809	28.38%
535	18.77%
738	25.89%
1209	42.41%
1077	37.78%
661	23.18%
446	15.64%
669	23.47%
2642	92.67%
1662	58.30%
1146	40.20%
380	13.33%
347	12.17%
388	13.61%
821	28.80%
684	23.99%
641	22.48%
1011	35.46%
890	31.22%
2721	95.44%
647	22.69%
361	12.66%

832	29.18%
1467	51.46%
801	28.10%
138	4.84%
1022	35.85%
1629	57.14%
897	31.46%
329	11.54%
34	1.19%
1434	50.30%
346	12.14%
1104	38.72%
292	10.24%
815	28.59%
819	28.73%
1270	44.55%
1144	40.13%
668	23.43%
1288	45.18%
1809	63.45%
1626	57.03%
722	25.32%
345	12.10%
843	29.57%
855	29.99%
1002	35.15%
196	6.87%
865	30.34%
737	25.85%
818	28.69%
377	13.22%
659	23.11%
415	14.56%
293	10.28%
678	23.78%
1281	44.93%
669	23.47%
1584	55.56%
857	30.06%
995	34.90%
997	34.97%
315	11.05%
882	30.94%
640	22.45%
673	23.61%

173	6.07%
367	12.87%
1555	54.54%
612	21.47%
1255	44.02%
937	32.87%
555	19.47%
111	3.89%
1155	40.51%
608	21.33%
1023	35.88%
893	31.32%
790	27.71%
1696	59.49%
865	30.34%
1215	42.62%
1529	53.63%
1175	41.21%
1461	51.25%
997	34.97%
978	34.30%
720	25.25%
165	5.79%
1406	49.32%
205	7.19%
770	27.01%
2753	96.56%
444	15.57%
827	29.01%
672	23.57%
123	4.31%
128	4.49%
865	30.34%
883	30.97%
357	12.52%
1314	46.09%

# of Background Sequences with Motif(of 47159)	% of Background Sequences with Motif
9893.9	20.98%
9825.2	20.83%
12345.5	26.18%
12501.5	26.51%
10548	22.37%
9618.1	20.39%
13734.4	29.12%
4262.5	9.04%
10910.9	23.14%
11547.2	24.49%
11440.8	24.26%
10918	23.15%
3418.4	7.25%
16488.6	34.96%
14064.1	29.82%
11815.5	25.05%
9673.8	20.51%
9949.3	21.10%
11369.3	24.11%
1104.9	2.34%
1005.5	2.13%
902.4	1.91%
9695.8	20.56%
12359.3	26.21%
12142.7	25.75%
4948.4	10.49%
6589.6	13.97%
20839.8	44.19%
12958.6	27.48%
13890.3	29.45%
11934.6	25.31%
6217.9	13.18%
13979.7	29.64%
9620.8	20.40%
27029.2	57.31%
14992.5	31.79%
3663.1	7.77%
1142.3	2.42%
16485.1	34.96%
10501.5	22.27%
15019.5	31.85%
26968.7	57.19%
4649	9.86%

15290.1	32.42%
11012.5	23.35%
19345.9	41.02%
8990.1	19.06%
13601.5	28.84%
19109.5	40.52%
18399.8	39.02%
14036.6	29.76%
23617.3	50.08%
15380.5	32.61%
18035.2	38.24%
11771.3	24.96%
16903.6	35.84%
10790.4	22.88%
7940.4	16.84%
15865.6	33.64%
16319.8	34.61%
7902.9	16.76%
9630.6	20.42%
7442.4	15.78%
15844.8	33.60%
15894.7	33.70%
5166	10.95%
12725.6	26.98%
6583.4	13.96%
11199.5	23.75%
5903.1	12.52%
5982	12.68%
14681	31.13%
19126.9	40.56%
4074.7	8.64%
15161.6	32.15%
10222.9	21.68%
24043.6	50.98%
3877.9	8.22%
1025.1	2.17%
7221.1	15.31%
8034.1	17.04%
5624.3	11.93%
21368	45.31%
6836.5	14.50%
5897	12.50%
15216.5	32.27%
14343.5	30.41%
26676.6	56.57%
15513.3	32.90%

6904.2	14.64%
7535.9	15.98%
10734.7	22.76%
39398.5	83.54%
18068.8	38.31%
27002.6	57.26%
6591.9	13.98%
17813.6	37.77%
7900.5	16.75%
18418.6	39.06%
14449.8	30.64%
11788.8	25.00%
8304.7	17.61%
13947.2	29.57%
14268.9	30.26%
19685	41.74%
20291.8	43.03%
7690.6	16.31%
8015.5	17.00%
13630.5	28.90%
11176.2	23.70%
18942.7	40.17%
6177.7	13.10%
11620.7	24.64%
7359.8	15.61%
10538.9	22.35%
18073.9	38.33%
15973.5	33.87%
9374.1	19.88%
6067.8	12.87%
9511.7	20.17%
42621.8	90.38%
25614.4	54.31%
17129.9	36.32%
5085.8	10.78%
4594.3	9.74%
5215.8	11.06%
11976.1	25.39%
9813.5	20.81%
9150.1	19.40%
15028.2	31.87%
13092.4	27.76%
44180	93.68%
9269.7	19.66%
4867.9	10.32%

12204.5	25.88%
22511.5	47.73%
11716	24.84%
1609.2	3.41%
15261.2	32.36%
25214.4	53.47%
13266.1	28.13%
4414.9	9.36%
267.2	0.57%
22052.2	46.76%
4697.9	9.96%
16667.5	35.34%
3887.1	8.24%
12040.7	25.53%
12107.3	25.67%
19404.1	41.15%
17349.7	36.79%
9733.7	20.64%
19728.7	41.83%
28372.8	60.16%
25315	53.68%
10604.3	22.49%
4730.7	10.03%
12537	26.58%
12731.4	27.00%
15107.4	32.03%
2511.8	5.33%
12906.9	27.37%
10865.6	23.04%
12167.5	25.80%
5242.5	11.12%
9645.3	20.45%
5833.7	12.37%
3971.9	8.42%
9952.4	21.10%
19685.3	41.74%
9815.3	20.81%
24689.3	52.35%
12817.9	27.18%
15039.1	31.89%
15074.4	31.96%
4328.8	9.18%
13248.4	28.09%
9388.1	19.91%
9913.1	21.02%

2211.1	4.69%
5149.1	10.92%
24287.4	51.50%
8978.8	19.04%
19344.3	41.02%
14183	30.07%
8096.2	17.17%
1340.2	2.84%
17735.2	37.61%
8941.8	18.96%
15601	33.08%
13502.5	28.63%
11852.9	25.13%
26684.3	56.58%
13063.8	27.70%
18745.8	39.75%
23919.2	50.72%
18096.3	38.37%
22832.1	48.41%
15249.2	32.34%
14942	31.68%
10792.3	22.88%
2157.6	4.58%
21946.3	46.54%
2754.8	5.84%
11615.4	24.63%
45020.6	95.46%
6460.9	13.70%
12563.3	26.64%
10079.4	21.37%
1565.2	3.32%
1641	3.48%
13193.1	27.98%
13486.2	28.60%
5126.4	10.87%
20523.6	43.52%

Supplementary Table 2

Motif Name (Size 1000)-Mature Adipocytes
CEBPB_MOUSE.H10MO.A
CEBPA_MOUSE.H10MO.B
CEBPD_MOUSE.H10MO.B
CEBP(bZIP)/CEBPb-ChIP-Seq(GSE21512)/Homer
DBP_MOUSE.H10MO.B
PPARG_MOUSE.H10MO.A
CEBPG_MOUSE.H10MO.C
PPARE(NR/DR1)/3T3L1-Pparg-ChIP-Seq(GSE13511)/Homer
CEBP:AP1(bZIP)/ThioMac-CEBPb-ChIP-Seq(GSE21512)/Homer
RXR(NR/DR1)/3T3L1-RXR-ChIP-Seq(GSE13511)/Homer
PPARA_MOUSE.H10MO.C
NR2C2_MOUSE.H10MO.A
HLF_MOUSE.H10MO.C
CEBPE_MOUSE.H10MO.A
COT1_MOUSE.H10MO.S
NFIA_MOUSE.H10MO.C
NF1(CTF)/LNCAP-NF1-ChIP-Seq(Unpublished)/Homer
Atf4(bZIP)/MEF-Atf4-ChIP-Seq(GSE35681)/Homer
NF1-halfsite(CTF)/LNCaP-NF1-ChIP-Seq(Unpublished)/Homer
PPARD_MOUSE.H10MO.D
COT2_MOUSE.H10MO.B
EBF(EBF)/proBcell-EBF-ChIP-Seq(GSE21978)/Homer
COE1_MOUSE.H10MO.C
HNF4A_MOUSE.H10MO.A
NR4A2_MOUSE.H10MO.C
PPARG_MOUSE.H10MO.S
COT2_MOUSE.H10MO.S
COT1_MOUSE.H10MO.B
ERR1_MOUSE.H10MO.A
Nur77(NR)/K562-NR4A1-ChIP-Seq(GSE31363)/Homer
Tlx?(NR)/NPC-H3K4me1-ChIP-Seq(GSE16256)/Homer
PPARA_MOUSE.H10MO.S
NR4A3_MOUSE.H10MO.D
Chop(bZIP)/MEF-Chop-ChIP-Seq(GSE35681)/Homer
NR2C1_MOUSE.H10MO.C
NR1H2_MOUSE.H10MO.D
TR4(NR/DR1)/Hela-TR4-ChIP-Seq(GSE24685)/Homer
Erra(NR)/HepG2-Erra-ChIP-Seq(GSE31477)/Homer

NR1D1_MOUSE.H10MO.D
HNF4a(NR/DR1)/HepG2-HNF4a-ChIP-Seq(GSE25021)/Homer
EBF1(EBF)/Near-E2A-ChIP-Seq(GSE21512)/Homer
Esrrb(NR)/mES-Esrrb-ChIP-Seq(GSE11431)/Homer

NFIA_MOUSE.H10MO.S
GRE/RAW264.7-GRE-ChIP-Seq(Unpublished)/Homer
NR2F6_MOUSE.H10MO.D
ERR2_MOUSE.H10MO.B
GRE(NR/IR3)/A549-GR-ChIP-Seq(GSE32465)/Homer
RARB_MOUSE.H10MO.D
ARE(NR)/LNCAP-AR-ChIP-Seq(GSE27824)/Homer
NR1I3_MOUSE.H10MO.C
RORA_MOUSE.H10MO.B
NFIL3_MOUSE.H10MO.C
ESR2_MOUSE.H10MO.S
NR4A1_MOUSE.H10MO.C
ERR3_MOUSE.H10MO.B
GFI1B_MOUSE.H10MO.C
HNF4G_MOUSE.H10MO.C
AMYB(HTH)/Testes-AMYB-ChIP-Seq(GSE44588)/Homer
Gfi1b(Zf)/HPC7-Gfi1b-ChIP-Seq(GSE22178)/Homer
BMYB(HTH)/Hela-BMYB-ChIPSeq(GSE27030)/Homer
PRGR_MOUSE.H10MO.C
GFI1_MOUSE.H10MO.C
Nr5a2(NR)/Pancreas-LRH1-ChIP-Seq(GSE34295)/Homer
ANDR_MOUSE.H10MO.B
Nr5a2(NR)/mES-Nr5a2-ChIP-Seq(GSE19019)/Homer
NR1I3_MOUSE.H10MO.S
RARA_MOUSE.H10MO.S
RARG_MOUSE.H10MO.S
VDR_MOUSE.H10MO.S
AP-2alpha(AP2)/Hela-AP2alpha-ChIP-Seq(GSE31477)/Homer
MEF2A_MOUSE.H10MO.B
AP-2gamma(AP2)/MCF7-TFAP2C-ChIP-Seq(GSE21234)/Homer
NR5A2_MOUSE.H10MO.A
AP2A_MOUSE.H10MO.A
MTF1_MOUSE.H10MO.C
GCR_MOUSE.H10MO.C
NR2E3_MOUSE.H10MO.C
NR1I2_MOUSE.H10MO.S
PR(NR)/T47D-PR-ChIP-Seq(GSE31130)/Homer
TLX1_MOUSE.H10MO.D
RXRB_MOUSE.H10MO.C
RORG_MOUSE.H10MO.C
Reverb(NR/DR2)/BLRP(RAW)-Reverba-ChIP-Seq(GSE45914)/Homer
Unknown6/Drosophila-Promoters/Homer
Mef2c(MADS)/GM12878-Mef2c-ChIP-Seq(GSE32465)/Homer
AARE(HLH)/mES-cMyc-ChIP-Seq/Homer

CEBP:CEBP(bZIP)/MEF-Chop-ChIP-Seq(GSE35681)/Homer

AP2C_MOUSE.H10MO.A
MEF2D_MOUSE.H10MO.C
ZEP2_MOUSE.H10MO.D
MEF2C_MOUSE.H10MO.B
THA_MOUSE.H10MO.S
STF1_MOUSE.H10MO.B
ZFX(Zf)/mES-Zfx-ChIP-Seq(GSE11431)/Homer
HRE(HSF)/Striatum-HSF1-ChIP-Seq(GSE38000)/Homer
PAX5_MOUSE.H10MO.S
MYB_MOUSE.H10MO.C
THA_MOUSE.H10MO.C
Tcf12(HLH)/GM12878-Tcf12-ChIP-Seq(GSE32465)/Homer
NR1I2_MOUSE.H10MO.C
NR6A1_MOUSE.H10MO.B
Ap4(HLH)/AML-Tfap4-ChIP-Seq(GSE45738)/Homer

RXRA_MOUSE.H10MO.C
P73_MOUSE.H10MO.D
CHR/Cell-Cycle-Exp/Homer
HNF1A_MOUSE.H10MO.A
FOXA1(Forkhead)/LNCAP-FOXA1-ChIP-Seq(GSE27824)/Homer
RXRG_MOUSE.H10MO.C
SUT1?/SacCer-Promoters/Homer
GAGA-repeat/SacCer-Promoters/Homer
AR-halfsite(NR)/LNCaP-AR-ChIP-Seq(GSE27824)/Homer
THB_MOUSE.H10MO.S
DLX3_MOUSE.H10MO.C
Rbpj1(?)/Panc1-Rbpj1-ChIP-Seq(GSE47459)/Homer
ZNF143|STAF(Zf)/CUTLL-ZNF143-ChIP-Seq(GSE29600)/Homer
LXRE(NR/DR4)/BLRP(RAW)-LXRb-ChIP-Seq(GSE21512)/Homer
HESX1_MOUSE.H10MO.D
NF2L2_MOUSE.H10MO.D
MEIS1_MOUSE.H10MO.C
SPZ1_MOUSE.H10MO.D
TRa(NR)/C17.2-TRa-ChIP-Seq(GSE38347)/Homer
ZNF711(Zf)/SH-SY5Y-ZNF711-ChIP-Seq(GSE20673)/Homer
SUH_MOUSE.H10MO.C
ISL1_MOUSE.H10MO.D
SOX4_MOUSE.H10MO.C
MyoD(HLH)/Myotube-MyoD-ChIP-Seq(GSE21614)/Homer
MYB(HTH)/ERMYB-Myb-ChIPSeq(GSE22095)/Homer
STAT2_MOUSE.H10MO.B
Initiator/Drosophila-Promoters/Homer
TEAD4_MOUSE.H10MO.D
FOXA1(Forkhead)/MCF7-FOXA1-ChIP-Seq(GSE26831)/Homer

RARG_MOUSE.H10MO.C

Consensus	P-value	Log P-value	q-value (Benjamini)
KATTGCAYAAT	1.00E-154	-3.56E+02	0
TATTKTGCAATA	1.00E-122	-2.83E+02	0
RRTTRBGCAAT	1.00E-115	-2.67E+02	0
ATTGCGCAAC	1.00E-102	-2.36E+02	0
RTTATGYAABM	1.00E-76	-1.75E+02	0
AASTRGGKCAAAGGTCA	1.00E-75	-1.75E+02	0
ATTKTGCAATCTB	1.00E-59	-1.37E+02	0
TGACCTTTGCCCCA	1.00E-54	-1.26E+02	0
DRTGTTGCAA	1.00E-52	-1.22E+02	0
TAGGGCAAAGGTCA	1.00E-51	-1.18E+02	0
AGGTCAAAGGTCA	1.00E-49	-1.15E+02	0
RGGTCAAAGGTCA	1.00E-49	-1.14E+02	0
GATTRYGTAAYMS	1.00E-40	-9.24E+01	0
TTGCGMAATCTY	1.00E-39	-9.18E+01	0
GKTCAAAGGTCA	1.00E-36	-8.43E+01	0
TTGGCANGNTGCCAR	1.00E-35	-8.27E+01	0
CYTGGCABNSTGCCAR	1.00E-35	-8.26E+01	0
MTGATGCAAT	1.00E-33	-7.80E+01	0
YTGCCAAG	1.00E-30	-7.09E+01	0
YAGGNBAAAGGTCA	1.00E-29	-6.68E+01	0
CAAAGGTCAA	1.00E-28	-6.66E+01	0
DGTCCCYRGGGA	1.00E-28	-6.65E+01	0
GTCCCTGGGGASY	1.00E-27	-6.39E+01	0
RGGBCAAAGTYCA	1.00E-27	-6.35E+01	0
AAAGGTCAS	1.00E-27	-6.25E+01	0
MAWAGGTCA	1.00E-26	-6.19E+01	0
RAGGTCAAAGGTCAA	1.00E-24	-5.59E+01	0
CAAAGGTCA	1.00E-23	-5.45E+01	0
YCAAGGTCA	1.00E-22	-5.14E+01	0
TGACCTTTNCNT	1.00E-21	-5.07E+01	0
CTGGCAGSCTGCCA	1.00E-21	-4.91E+01	0
AAGGTCA	1.00E-20	-4.62E+01	0
CAAAGGTCAS	1.00E-19	-4.57E+01	0
ATTGCATCAT	1.00E-19	-4.55E+01	0
GSCCAGAGGTCAB	1.00E-19	-4.43E+01	0
TAAAGGTCAAAGGTCAACG	1.00E-18	-4.28E+01	0
GAGGTCAAAGGTCA	1.00E-18	-4.24E+01	0
CAAAGGTCAG	1.00E-17	-4.13E+01	0
ARGDVMVWAGGTCAGDGGG	1.00E-16	-3.80E+01	0
CARRGKBCAAAGTYCA	1.00E-16	-3.69E+01	0
GTCCCCWGGGGA	1.00E-15	-3.65E+01	0
KTGACCTTGA	1.00E-15	-3.49E+01	0

MTTGGCA	1.00E-14	-3.45E+01	0
VAGRACAKWCTGTYC	1.00E-13	-3.14E+01	0
AGGWCAAAGKTCANNKGA	1.00E-13	-3.07E+01	0
TCAAGGTCA	1.00E-13	-3.01E+01	0
NRGVACABNVTGTYCY	1.00E-12	-2.90E+01	0
SAGGTCAGRRV	1.00E-12	-2.82E+01	0
RGRACASNSTGTYCYB	1.00E-11	-2.69E+01	0
GRGGTCARRRAAGTTCAK	1.00E-10	-2.52E+01	0
AWAASTAGGTCA	1.00E-10	-2.52E+01	0
GTTAYGTAAYKYMK	1.00E-10	-2.48E+01	0
AGGTCAS	1.00E-09	-2.24E+01	0
RAAGGTCAS	1.00E-09	-2.20E+01	0
TCAAGGTCA	1.00E-09	-2.12E+01	0
GCTGWGATTTV	1.00E-09	-2.07E+01	0
GGCCAAAGTCCA	1.00E-08	-2.02E+01	0
TGGCAGTTGG	1.00E-08	-2.00E+01	0
MAATCACTGC	1.00E-08	-1.96E+01	0
NHAACBGYYV	1.00E-08	-1.95E+01	0
RGAACANYCTGTH	1.00E-08	-1.86E+01	0
GCWGTGATTT	1.00E-08	-1.85E+01	0
BTCAAGGTCA	1.00E-07	-1.68E+01	0
GGWACABVRTGTTCTT	1.00E-07	-1.65E+01	0
BTCAAGGTCA	1.00E-07	-1.64E+01	0
RAGTTCAB	1.00E-07	-1.64E+01	0
SAGGTCA	1.00E-06	-1.61E+01	0
GAGGTCA	1.00E-06	-1.61E+01	0
GRGKTCA	1.00E-06	-1.61E+01	0
ATGCCCTGAGGC	1.00E-06	-1.56E+01	0
RDKCTATTTWTAGM	1.00E-06	-1.55E+01	0
SCCTSAGGSCAW	1.00E-06	-1.55E+01	0
YTCAAGGTCAN	1.00E-06	-1.54E+01	0
GCCTGRGGC	1.00E-06	-1.45E+01	0
MGKGCCGTGTGCAAARS	1.00E-06	-1.42E+01	0
GAGAACAKWVWGTNCHDR	1.00E-05	-1.31E+01	0
AAAGTCAAAAGTCA	1.00E-05	-1.29E+01	0
RAGTTCAB	1.00E-05	-1.25E+01	0
VAGRACAKNCTGTBC	1.00E-05	-1.23E+01	0
CTGGYAMBDDGCCAAGR	1.00E-05	-1.21E+01	0.0001
TSAGGTCACR	1.00E-05	-1.19E+01	0.0001
DWAAGTRGGTCAG	1.00E-05	-1.17E+01	0.0001
GTRGGTCASTGGGTCA	1.00E-05	-1.17E+01	0.0001
AATTTTAAAA	1.00E-04	-1.09E+01	0.0002
DCYAAAAATAGM	1.00E-04	-1.05E+01	0.0002
GATTGCATCA	1.00E-04	-1.03E+01	0.0003
NTNATGCAAYMNNHTGMAAY	1.00E-04	-1.01E+01	0.0003

GCCTGRGGC	1.00E-04	-1.00E+01	0.0004
GCTATTTTTAGV	1.00E-04	-9.76E+00	0.0005
GGTDGGGWAACCCS	1.00E-04	-9.54E+00	0.0006
TKCTAWAAATAGM	1.00E-04	-9.51E+00	0.0006
YTCAGGTCAS	1.00E-04	-9.38E+00	0.0007
YCAAGGTCA	1.00E-04	-9.25E+00	0.0008
AGGCCTRG	1.00E-03	-9.11E+00	0.0009
TTCTAGAABNTTCTA	1.00E-03	-9.09E+00	0.0009
GGCTGAGG	1.00E-03	-8.72E+00	0.0012
GTGGCAGTTGG	1.00E-03	-8.68E+00	0.0013
RGGTCABTTCAGGTCAG	1.00E-03	-8.40E+00	0.0017
VCAGCTGYTG	1.00E-03	-8.37E+00	0.0017
DDRGGTCAANNAAGTTCAB	1.00E-03	-8.28E+00	0.0018
AAGTTCAAGGTCA	1.00E-03	-8.15E+00	0.0021
NAHCAGCTGD	1.00E-03	-8.06E+00	0.0023
RGGRTCARRAGTTCAAGGT CAG	1.00E-03	-8.06E+00	0.0023
GGGGCADGYYYB	1.00E-03	-8.05E+00	0.0023
SRGTTTCAAA	1.00E-03	-7.98E+00	0.0024
RGTTAATAATTAACM	1.00E-03	-7.87E+00	0.0026
WAAGTAAACA	1.00E-03	-7.84E+00	0.0027
RAGTTCAAGGTCAGCCT	1.00E-03	-7.81E+00	0.0027
CCCCGCGC	1.00E-03	-7.62E+00	0.0033
CTYTCTYTCTCTCTC	1.00E-03	-7.55E+00	0.0035
CCAGGAACAG	1.00E-03	-7.55E+00	0.0035
SGTCAGGTCA	1.00E-03	-7.44E+00	0.0038
GMTAATTACW	1.00E-03	-7.32E+00	0.0043
HTTTCCCASG	1.00E-03	-7.32E+00	0.0043
ATTTCCCAGVAKSCY	1.00E-03	-7.27E+00	0.0044
RGGTTACTANAGGTCA	1.00E-03	-7.27E+00	0.0044
ABSYGGCWCGTRRCMT	1.00E-03	-7.21E+00	0.0046
VRTGACTCAGCA	1.00E-03	-7.20E+00	0.0046
TGACAGYTTTAHG	1.00E-03	-7.17E+00	0.0047
CGGCTGTWACCBYGGG	1.00E-03	-7.12E+00	0.0049
GGTCANYTGAGGWCA	1.00E-03	-7.00E+00	0.0055
AGGCCTAG	1.00E-03	-6.99E+00	0.0055
CGTGGGAAA	1.00E-02	-6.84E+00	0.0063
SYTAATR	1.00E-02	-6.84E+00	0.0063
CSCTTTGTTCTC	1.00E-02	-6.80E+00	0.0065
RRCAGCTGYTSY	1.00E-02	-6.74E+00	0.0068
GGCVGTTR	1.00E-02	-6.67E+00	0.0073
TCAGTTTCNKTTTCC	1.00E-02	-6.65E+00	0.0074
NTCAGTYG	1.00E-02	-6.62E+00	0.0075
AAAAATAKCYCT	1.00E-02	-6.43E+00	0.0091
WAAGTAAACA	1.00E-02	-6.41E+00	0.0091

GGRGKTCAVCNAVAGGTCAV	1.00E-02	-6.31E+00	0.0101

# of Target Sequences with Motif(of 3228)	% of Target Sequences with Motif
958	29.68%
1306	40.46%
958	29.68%
1416	43.87%
912	28.25%
1825	56.54%
974	30.17%
1714	53.10%
1386	42.94%
1855	57.47%
1446	44.80%
1553	48.11%
491	15.21%
645	19.98%
1306	40.46%
1076	33.33%
743	23.02%
570	17.66%
2234	69.21%
1266	39.22%
1183	36.65%
446	13.82%
1227	38.01%
1438	44.55%
1203	37.27%
1052	32.59%
1379	42.72%
1183	36.65%
1165	36.09%
424	13.14%
806	24.97%
791	24.50%
1173	36.34%
419	12.98%
1122	34.76%
1030	31.91%
294	9.11%
2564	79.43%
1630	50.50%
917	28.41%
1418	43.93%
1138	35.25%

753	23.33%
472	14.62%
1448	44.86%
1097	33.98%
285	8.83%
1076	33.33%
564	17.47%
996	30.86%
796	24.66%
452	14.00%
584	18.09%
1324	41.02%
870	26.95%
1049	32.50%
1019	31.57%
1948	60.35%
1027	31.82%
1889	58.52%
1149	35.59%
950	29.43%
1214	37.61%
781	24.19%
965	29.89%
874	27.08%
665	20.60%
665	20.60%
665	20.60%
934	28.93%
1038	32.16%
1171	36.28%
1189	36.83%
728	22.55%
416	12.89%
1444	44.73%
1535	47.55%
814	25.22%
2592	80.30%
666	20.63%
1052	32.59%
796	24.66%
250	7.74%
1356	42.01%
724	22.43%
151	4.68%
254	7.87%

844	26.15%
894	27.70%
526	16.29%
794	24.60%
961	29.77%
1088	33.71%
1533	47.49%
406	12.58%
1221	37.83%
615	19.05%
1004	31.10%
1196	37.05%
914	28.31%
1293	40.06%
1553	48.11%
1593	49.35%
809	25.06%
970	30.05%
824	25.53%
1820	56.38%
1443	44.70%
3041	94.21%
3130	96.96%
3058	94.73%
839	25.99%
509	15.77%
1859	57.59%
631	19.55%
98	3.04%
473	14.65%
867	26.86%
856	26.52%
370	11.46%
614	19.02%
1832	56.75%
961	29.77%
298	9.23%
1023	31.69%
974	30.17%
2035	63.04%
1963	60.81%
2528	78.31%
1137	35.22%
1588	49.19%

957	29.65%

# of Background Sequences with Motif(of 46649)	% of Background Sequences with Motif
5653.8	12.12%
10225.4	21.93%
6531	14.01%
12250.8	26.27%
7182.2	15.40%
18826.5	40.37%
8520.1	18.27%
18397.5	39.45%
14038.4	30.11%
20611	44.20%
15007.9	32.18%
16470.8	35.32%
3775.4	8.10%
5498	11.79%
13948	29.91%
10973.2	23.53%
6831	14.65%
4895.9	10.50%
27656.9	59.31%
13941.3	29.90%
12839.4	27.53%
3706.7	7.95%
13512.4	28.98%
16397	35.16%
13236.1	28.38%
11256.6	24.14%
15854.9	34.00%
13242.6	28.40%
13110.5	28.12%
3763.9	8.07%
8474.4	18.17%
8374.4	17.96%
13432.5	28.81%
3818	8.19%
12797.8	27.44%
11621.2	24.92%
2458.3	5.27%
33928	72.76%
20127.6	43.16%
10349.2	22.19%
17203.1	36.89%
13406.8	28.75%

8289.5	17.78%
4825.8	10.35%
17909.4	38.41%
13073.4	28.04%
2638.6	5.66%
12879.5	27.62%
6137	13.16%
11937	25.60%
9245.6	19.83%
4793.3	10.28%
6576.6	14.10%
16659.3	35.73%
10435.4	22.38%
12908.5	27.68%
12525.2	26.86%
25745.1	55.21%
12671.4	27.17%
24914.6	53.43%
14427.3	30.94%
11683.2	25.05%
15450.4	33.13%
9499	20.37%
12019.7	25.78%
10770.7	23.10%
7955.2	17.06%
7955.2	17.06%
7955.2	17.06%
11643.5	24.97%
13087.6	28.07%
14945.5	32.05%
15202.4	32.60%
8898.3	19.08%
4763.5	10.22%
18989.5	40.72%
20305.7	43.55%
10205.1	21.88%
35927.3	77.05%
8215.3	17.62%
13548.1	29.05%
10017.5	21.48%
2739.9	5.88%
17925.2	38.44%
9114.8	19.55%
1554.6	3.33%
2856.2	6.13%

10811.1	23.18%
11526.3	24.72%
6489.2	13.92%
10161	21.79%
12494.5	26.79%
14287.8	30.64%
20630.3	44.24%
4906.3	10.52%
16218.2	34.78%
7763.7	16.65%
13188.2	28.28%
15900.9	34.10%
11938.9	25.60%
17300.9	37.10%
21029.3	45.10%
21605.5	46.33%
10495.2	22.51%
12752.7	27.35%
10721.5	22.99%
24912.5	53.42%
19479.7	41.77%
43241.6	92.73%
44694.5	95.85%
43518.9	93.33%
10971.1	23.53%
6422.3	13.77%
25539.8	54.77%
8100.6	17.37%
1007.1	2.16%
5940.8	12.74%
11387.5	24.42%
11236.6	24.10%
4553.9	9.77%
7890.3	16.92%
25185.2	54.01%
12743.2	27.33%
3608.3	7.74%
13621.1	29.21%
12937.6	27.74%
28183.6	60.44%
27134.5	58.19%
35470.3	76.07%
15277.4	32.76%
21728.2	46.60%

12746.1	27.33%

Supplementary Table 3-Preadipocytes

Motif.Name (Cluster 1)	Consensus	P.value	Log.P.value
NFIA_MOUSE.H10MO.C	TTGGCANGNTGCCAR	1.00E-15	-36.24
NFIA_MOUSE.H10MO.S	MTTGGCA	1.00E-11	-25.47
CEBPA_MOUSE.H10MO.B	TATTKTGCAATA	1.00E-07	-17.76
DBP_MOUSE.H10MO.B	RTTATGYAABM	1.00E-05	-12.66
BCL6_MOUSE.H10MO.C	WTTCCTRGAAAGC	1.00E-04	-11.47
ELF3_MOUSE.H10MO.D	GGSAAACAGGAARY	0.001	-7.178
PPARG_MOUSE.H10MO.S	MAWAGGTCA	0.1	-4.463
PPARA_MOUSE.H10MO.C	AGGTCAAAGGTCA	0.1	-3.288
PPARA_MOUSE.H10MO.S	AAGGTCA	0.1	-3.061
IRF4_MOUSE.H10MO.C	AAAARAGRAAVTGAAA	0.1	-2.484
KLF15_MOUSE.H10MO.D	RSCKGGGAGGTGGGGSS	0.1	-2.373
PPARG_MOUSE.H10MO.A	AASTRGGKCAAAGGTCA	1	-1.437
SOX18_MOUSE.H10MO.D	GHWHVMATTGTTBTYKTCC	1	-0.6563
RREB1_MOUSE.H10MO.D	DKGGKKGKGGGTGKTTTGGGGT	1	-0.1514
RXRG_MOUSE.H10MO.C	RAGTTCAAGGTCAGCCT	1	-3.40E-05

Motif.Name (Cluster 2)	Consensus	P.value	Log.P.value
ETV4_MOUSE.H10MO.B	SAGGAAGT	1.00E-11	-25.75
IRF7_MOUSE.H10MO.C	GAAASYGAAA	1.00E-05	-13.13
KLF4_MOUSE.H10MO.C	WGGGYGKGGCCS	0.001	-8.386
MAFB_MOUSE.H10MO.D	TGCTGACDS	0.01	-6.476
HNF1B_MOUSE.H10MO.B	GGTTAATRATTAAC	0.1	-3.401
IRF9_MOUSE.H10MO.C	GAAAGCGAAACT	0.1	-3.325
ARNT2_MOUSE.H10MO.D	GNCGTGGSAGRC	1	-1.567

Motif.Name (Cluster 3)	Consensus	P.value	Log.P.value
FOSL1_MOUSE.H10MO.C	DRTGACTCAYN	1.00E-278	-640.5
TEAD4_MOUSE.H10MO.D	AAAAATAKCYCT	1.00E-10	-24.89
CEBPB_MOUSE.H10MO.A	KATTGCAYAAT	1.00E-08	-19.75
RUNX3_MOUSE.H10MO.B	KGGGKTTTGTGGTT	1	-1.997

DLX3_MOUSE.H10MO.C	GMTAATTACW	1	-0.5721
MYC_MOUSE.H10MO.A	GSRASCACGTGG	1	-0.03625
SNAI1_MOUSE.H10MO.C	CCAGGTGG	1	0

Motif.Name (Cluster 4)	Consensus	P.value	Log.P.value
FOSL2_MOUSE.H10MO.A	GGATGAGTCAY	1.00E-276	-637.8
JUNB_MOUSE.H10MO.B	DVTGAGTCAK	1.00E-240	-554.1
RUNX1_MOUSE.H10MO.B	TYTGTGGTTA	1.00E-18	-42.19
ATF3_MOUSE.H10MO.A	GTGACGYMA	1.00E-05	-11.61
FOXD1_MOUSE.H10MO.D	CWWTGTTTACHTAA	0.001	-7.737
NFIL3_MOUSE.H10MO.C	GTTAYGTAAYKYMK	0.01	-5.828
ETS2_MOUSE.H10MO.C	SMVGGAAGTGG	0.01	-5.324
NR4A2_MOUSE.H10MO.C	AAAGGTCAS	0.01	-5.131
GATA2_MOUSE.H10MO.A	RSAGATAAGRR	0.1	-4.393
FOXC2_MOUSE.H10MO.D	GTTTGTTTTGYCAGA	0.1	-4.363
FOXF2_MOUSE.H10MO.D	TGTTTACHTWD	0.1	-3.99
VDR_MOUSE.H10MO.S	GRGKTCA	0.1	-3.858
NR4A3_MOUSE.H10MO.D	CAAAGGTCAS	0.1	-2.985
CREM_MOUSE.H10MO.C	CRVTGACGTCA	1	-2.07
HMGA2_MOUSE.H10MO.D	AATAWYSGSSAATAT	1	-1.147
EGR2_MOUSE.H10MO.C	DGCGTGGGCGG	1	-1.062
DLX2_MOUSE.H10MO.D	ATAATTAB	1	-0.68
SOX13_MOUSE.H10MO.D	YATTGTTY	1	-0.3804
NR4A1_MOUSE.H10MO.C	RAAGGTCAS	1	-0.3802
VDR_MOUSE.H10MO.C	GGGTCAWNGRGTTC	1	-0.3278
PITX2_MOUSE.H10MO.D	TKGGATTAMH	1	-2.00E-06

q.value..Benjamini.	X..of.Target.Sequences.with.Motif.of.2851.	X..of.Target.Sequences.with.Motif
0	822	28.83%
0	617	21.64%
0	775	27.18%
0	535	18.77%
1.00E-04	1146	40.20%
0.003	1155	40.51%
0.0335	733	25.71%
0.0903	913	32.02%
0.1065	552	19.36%
0.1715	2273	79.73%
0.1885	1154	40.48%
0.416	1140	39.99%
0.7793	841	29.50%
1	1501	52.65%
1	1042	36.55%

q.value..Benjamini	X..of.Target.Sequences.with.Motif.of.285	X..of.Target.Sequences.with.Mot
0	1161	40.72%
0	1267	44.44%
0.001	865	30.34%
0.0056	720	25.25%
0.0825	650	22.80%
0.0878	903	31.67%
0.3696	518	18.17%

q.value..Benjamini	X..of.Target.Sequences.with.Motif.of.285	X..of.Target.Sequences.with.Mot
0	1515	53.14%
0	1128	39.57%
0	464	16.27%
0.2568	812	28.48%

0.8304	417	14.63%
1	608	21.33%
1	494	17.33%

q.value..Benjamini	X..of.Target.Sequences.with.Motif.of.285	X..of.Target.Sequences.with.Mot
0	1439	50.47%
0	1526	53.53%
0	933	32.73%
1.00E-04	446	15.64%
0.0018	882	30.94%
0.01	357	12.52%
0.016	899	31.53%
0.0192	852	29.88%
0.0356	655	22.97%
0.0363	1108	38.86%
0.0506	1277	44.79%
0.0556	510	17.89%
0.1132	851	29.85%
0.2437	364	12.77%
0.5305	401	14.07%
0.5674	779	27.32%
0.7626	394	13.82%
0.9463	489	17.15%
0.9463	999	35.04%
0.9758	521	18.27%
1	656	23.01%

X..of.Background.Sequences.with.Motif.of.47159.	X..of.Background.Sequences.with.Motif
10501.5	22.27%
7902.9	16.76%
10734.7	22.76%
7359.8	15.61%
17129.9	36.32%
17735.2	37.61%
11255.2	23.87%
14367.8	30.47%
8549.8	18.13%
37091.6	78.65%
18511	39.25%
18541.2	39.32%
13923.8	29.52%
25295.5	53.64%
18945.2	40.17%

X..of.Background.Sequences.with.Motif.of.471	X..of.Background.Sequences.with.Mo
16319.8	34.61%
18942.7	40.17%
12906.9	27.37%
10792.3	22.88%
10077	21.37%
14197.8	30.11%
8288.3	17.57%

X..of.Background.Sequences.with.Motif.of.471	X..of.Background.Sequences.with.Mo
10548	22.37%
15844.8	33.60%
5897	12.50%
12989.9	27.54%

6942.3	14.72%
10713.6	22.72%
12465.9	26.43%

X..of.Background.Sequences.with.Motif.of.471	X..of.Background.Sequences.with.Mo
9618.1	20.39%
11547.2	24.49%
11934.6	25.31%
6067.8	12.87%
13248.4	28.09%
5126.4	10.87%
13818	29.30%
13083.2	27.74%
10010	21.23%
17366	36.82%
20202	42.84%
7757.7	16.45%
13413.1	28.44%
5683.6	12.05%
6482.1	13.75%
12723.8	26.98%
6516.9	13.82%
8242.3	17.48%
16719.4	35.45%
8812.4	18.69%
12608.9	26.74%

›tif

›tif

›tif

Supplementary Table 3-Mature Adipocytes

Motif.Name(Cluster 1)	Consensus	P.value
CEBPA_MOUSE.H10MO.B	TATTKTGCAATA	1.00E-122
DBP_MOUSE.H10MO.B	RTTATGYAABM	1.00E-76
PPARG_MOUSE.H10MO.A	AASTRGGKCAA AGGTCA	1.00E-75
PPARA_MOUSE.H10MO.C	AGGTCAAAGGT CA	1.00E-49
NFIA_MOUSE.H10MO.C	TTGGCANGNTG CCAR	1.00E-35
PPARG_MOUSE.H10MO.S	MAWAGGTCA	1.00E-26
PPARA_MOUSE.H10MO.S	AAGGTCA	1.00E-20
NFIA_MOUSE.H10MO.S	MTTGGCA	1.00E-14
RXRG_MOUSE.H10MO.C	RAGTTCAAGGT CAGCCT	0.001
SOX18_MOUSE.H10MO.D	GHWHVMATTGT TBTYKTCC	0.1
IRF4_MOUSE.H10MO.C	AAAARAGRAAV TGAAA	1
KLF15_MOUSE.H10MO.D	RSCKGGGAGGT GGGGSS	1
RREB1_MOUSE.H10MO.D	DKGGKKGKGG GTGKTTTGGGG T	1
ELF3_MOUSE.H10MO.D	GGSAAACAGGA ARY	1
BCL6_MOUSE.H10MO.C	WTTCCTRGAAA GC	1

Motif.Name(Cluster 2)	Consensus	P.value
HNF1B_MOUSE.H10MO.B	GGTTAATRATTAA	0.1
IRF7_MOUSE.H10MO.C	GAAASYGAAA	0.1
IRF9_MOUSE.H10MO.C	GAAAGCGAAACT	1
KLF4_MOUSE.H10MO.C	WGGGYGKGGCC	1
MAFB_MOUSE.H10MO.D	TGCTGACDS	1
ARNT2_MOUSE.H10MO.D	GNCGTGGSAGR	1
ETV4_MOUSE.H10MO.B	SAGGAAGT	1

Motif.Name(Cluster 3)	Consensus	P.value
CEBPB_MOUSE.H10MO.A	KATTGCAYAAT	1.00E-154
DLX3_MOUSE.H10MO.C	GMTAATTACW	0.001
TEAD4_MOUSE.H10MO.D	AAAAATAKCYCT	0.01
FOSL1_MOUSE.H10MO.C	DRTGACTCAYN	1
MYC_MOUSE.H10MO.A	GSRASCACGTGC	1

RUNX3_MOUSE.H10MO.B	KGGGKTTTGTGG	1
SNAI1_MOUSE.H10MO.C	CCAGGTGG	1

Motif.Name(Cluster 4)	Consensus	P.value
NR4A2_MOUSE.H10MO.C	AAAGGTCAS	1.00E-27
NR4A3_MOUSE.H10MO.D	CAAAGGTCAS	1.00E-19
NFIL3_MOUSE.H10MO.C	GTTAYGTAAYKYM	1.00E-10
NR4A1_MOUSE.H10MO.C	RAAGGTCAS	1.00E-09
VDR_MOUSE.H10MO.S	GRGKTCA	1.00E-06
FOXF2_MOUSE.H10MO.D	TGTTTACHTWD	0.01
ATF3_MOUSE.H10MO.A	GTGACGYMA	0.1
FOXD1_MOUSE.H10MO.D	CWWTGTTTACHT	0.1
FOXC2_MOUSE.H10MO.D	GTTTGTTTTGYCA	0.1
PITX2_MOUSE.H10MO.D	TKGGATTAMH	0.1
HMGA2_MOUSE.H10MO.D	AATAWYSGSSAAT	1
JUNB_MOUSE.H10MO.B	DVTGAGTCAK	1
RUNX1_MOUSE.H10MO.B	TYTGTGGTTA	1
VDR_MOUSE.H10MO.C	GGGTCAWNGRG	1
CREM_MOUSE.H10MO.C	CRVTGACGTCA	1
DLX2_MOUSE.H10MO.D	ATAATTAB	1
SOX13_MOUSE.H10MO.D	YATTGTTY	1
FOSL2_MOUSE.H10MO.A	GGATGAGTCAY	1
ETS2_MOUSE.H10MO.C	SMVGGAAGTGG	1
EGR2_MOUSE.H10MO.C	DGCGTGGGCGG	1
GATA2_MOUSE.H10MO.A	RSAGATAAGRR	1

Log.P.value	q.value..Benjamini.	X..of.Target.Sequences.with.Motif.of.3228.
-282.9	0	1306
-175.3	0	912
-174.5	0	1825
-114.9	0	1446
-82.74	0	1076
-61.92	0	1052
-46.21	0	791
-34.52	0	753
-7.808	0.0027	1443
-2.827	0.2118	957
-0.4276	1	2459
-0.08465	1	1318
-0.01029	1	1740
-0.007744	1	1131
-0.000233	1	1040

Log.P.value	q.value..Benjamini	X..of.Target.Sequences.with.Motif.of.322
-3.12	0.1669	715
-2.539	0.2656	1291
-1.423	0.6043	930
-1.15	0.722	965
-0.1839	1	723
-0.02346	1	569
-0.00012	1	1000

Log.P.value	q.value..Benjamini	X..of.Target.Sequences.with.Motif.of.322
-355.6	0	958
-7.317	0.0043	509
-6.429	0.0091	1137
-1.728	0.4881	728
-0.4428	1	742

-0.4393	1	915
0	1	781

Log.P.value	q.value..Benjamini	X..of.Target.Sequences.with.Motif.of.322
-62.51	0	1203
-45.69	0	1173
-24.77	0	452
-21.96	0	1324
-16.11	0	665
-5.753	0.0169	1428
-3.873	0.0893	447
-3.312	0.1408	927
-2.84	0.2101	1231
-2.653	0.2426	900
-2.24	0.3308	424
-1.302	0.6499	796
-1.046	0.7783	852
-0.8118	0.8906	629
-0.6512	0.9666	386
-0.6339	0.9755	418
-0.5731	1	533
-0.5551	1	649
-0.05356	1	909
-0.001239	1	842
-2.00E-06	1	565

X..of.Target.Sequences.with.Motif	X..of.Background.Sequences.with.Motif.of.46649.
40.46%	10225.4
28.25%	7182.2
56.54%	18826.5
44.80%	15007.9
33.33%	10973.2
32.59%	11256.6
24.50%	8374.4
23.33%	8289.5
44.70%	19479.7
29.65%	13238.8
76.18%	35650.3
40.83%	19598.9
53.90%	26074.7
35.04%	17288.3
32.22%	16379.7

X..of.Target.Sequences.with.Mot	X..of.Background.Sequences.with.Motif.of.466
22.15%	9750.2
39.99%	18077.3
28.81%	13168
29.89%	13755.9
22.40%	10770.7
17.63%	8849
30.98%	15856.1

X..of.Target.Sequences.with.Mot	X..of.Background.Sequences.with.Motif.of.466
29.68%	5653.8
15.77%	6422.3
35.22%	15277.4
22.55%	10196.2
22.99%	10839.3

28.35%	13349.8
24.19%	13320.5

X..of.Target.Sequences.with.Mot	X..of.Background.Sequences.with.Motif.of.466
37.27%	13236.1
36.34%	13432.5
14.00%	4793.3
41.02%	16659.3
20.60%	7955.2
44.24%	19514.1
13.85%	5889.6
28.72%	12726.9
38.14%	17154.1
27.88%	12458.8
13.14%	5780.8
24.66%	11279.6
26.39%	12164.3
19.49%	9035.5
11.96%	5585.6
12.95%	6055
16.51%	7743
20.11%	9431.5
28.16%	13731
26.08%	13268.1
17.50%	9658.3

X..of.Background.Sequences.with.M
otif

21.93%
15.40%

40.37%

32.18%

23.53%
24.14%
17.96%
17.78%

41.77%

28.39%

76.45%

42.03%

55.92%

37.07%

35.13%

X..of.Background.Sequences.with.Motif

20.91%
38.77%
28.24%
29.50%
23.10%
18.98%
34.00%

X..of.Background.Sequences.with.Motif

12.12%
13.77%
32.76%
21.87%
23.24%

28.63%
28.57%

X..of.Background.Sequences.with.Motif
28.38%
28.81%
10.28%
35.73%
17.06%
41.85%
12.63%
27.29%
36.79%
26.72%
12.40%
24.19%
26.09%
19.38%
11.98%
12.98%
16.60%
20.23%
29.45%
28.45%
20.71%

Supplementary Table 4

	shRNA	TRCN
Elf3	1	TRCN0000054415
Elf3	2	TRCN0000054417
Dbp	1	TRCN0000086025
Dbp	2	TRCN0000086026
Sox18	1	TRCN0000085710
Sox18	2	TRCN0000085711
Sox18	3	TRCN0000085712
Zfp467	1	TRCN0000085855
Zfp467	2	TRCN0000085856
Zfp467	3	TRCN0000085857
Hoxa4	2	TRCN0000070791
Hoxa4	3	TRCN0000070792
Ppara	1	TRCN0000026028
Ppara	2	TRCN0000026041
Ybx2	1	TRCN0000095321
Nfia	1	TRCN0000012085
Nfia	2	TRCN0000012087
Nfia	3	TRCN0000012084

Supplementary Figure 1

(A) Overview of the total number of genes expressed, differentially expressed and not expressed during differentiation.

(B) Hierarchical clustering (voom normalized counts, distance: correlation, cluster method: complete) of mRNA-seq time points and replicates.

(C) Clusters showing two of a total of four distinct patterns of gene expression belonging to Clusters 2 and 3.

(D) Gene ontology (GO) terms associated with genes from Cluster 2 and Cluster 3 against a background of all genes expressed during BFC differentiation (see Methods)

(E) Distribution of Pearson product-moment correlation coefficient of the expression level of all Cluster 2 and 3 TFs, as well as controls (all TFs) with *Ucp1* expression in the BAT of BXD mouse reference panel strains (top) and human clonal BAT-derived cell lines (bottom).

Supplementary Figure 2

(A) Hierarchical clustering (distance: correlation, cluster method: complete) of all H3K27ac time points and replicates based on H3K27ac levels (voom normalized counts, see Methods) at all differentially acetylated (DA) regions.

(B) Heatmaps visualizing H3K27ac ChIP-seq enrichment at regions specific for individual time-points in a 10kb window.

(C) Distribution of H3K27ac ChIP-seq enrichment within a 10kb region around the transcription start site (TSS) of differentially expressed genes (DEGs) from each cluster. The region around the TSS is partitioned into three compartments: a red triangle represents a region from +4 to +5kb of the TSS, a black dot represents a region from +1 to -1kb around the TSS and a green triangle represents a -4 to -5 kb of the TSS.

(D) UCSC browser screen shots of representative genes from each gene expression cluster: Cluster 1: *Ucp1* and *Pparg*, Cluster 2: *Klf4* and Cluster 3: *Tead4*.

(E) Overview of the differentially H3K27ac-enriched regions in Pre- versus Mature-adipocytes.

(F) Genomic distribution of the differentially H3K27ac-enriched regions, with regions annotated to TSS within a region of -1kb to +100bp (see Methods)

(G) GO term associated with genes in the proximity of Pre- and Mature adipocyte H3K27ac enriched regions.

Supplementary Figure 3

(A) Diagnostic scatter plot indicating the correlation between qPCR fold change (FC) versus barcoded mRNA seq FC (relative to Scrambled-I). Pearson's correlation coefficient between the two methods for all the samples is 0.99.

(B) Expression value of all the nodes in the network (Fig **6**) and their clustering pattern with respect to *Nfia* knockdown.

(C) Top overrepresented motifs in either Pre- or Mature adipocyte H3K27ac regions containing an NFI peak to detect co-occurring TFs that co-occur with NFIA.

Supplementary Figure 4

(A) Western blot film exposed for 30 seconds (B) and 10 seconds, showing protein expression levels of UCP1, PPARγ (both PPARγ1 and PPARγ2 isoforms indicated) and endogenous control α-TUBULIN at different time points during differentiation, specifically, Day 0, Day 4, and at Day 6 with and without treatment with the β-adrenergic stimulator, isoproterenol. Two biological replicates are shown in the film of which replicate 1 was chosen for the main figure (Fig 1C).

Supplementary Table 1

List of transcription factors and their membership values in the four identified clusters.

Supplementary Table 2

List of motifs enriched in Pre- and Mature-adipocyte specific differentially acetylated regions.

Supplementary Table 3

Motifs of transcription factors identified in the four different clusters and their motif enrichment scores in Pre- and Mature-adipocyte specific differentially acetylated regions.

Supplementary Table 4

List of short hairpin RNAs used for performing loss-of-function assays (from the Sigma library)
